# Self-amplifying RNA therapy encoding CNTF with disulfiram co-delivery promotes optic nerve repair through microglial pyroptosis inhibition and RGC axonal regeneration

**DOI:** 10.1186/s12951-026-04272-x

**Published:** 2026-03-13

**Authors:** Qianyue Zhang, Yusha Liu, Qin Wei, Mingyang Song, Siwei Liu, Haiyang Zhang, Tingyu Deng, Chutong Zhang, Kexin Tan, Rui Huang, Ni Ni, Jun Zhang, Ping Gu, Gang Du, Jipeng Li, Yingzhi Chen, Huifang Zhou, Xianqun Fan

**Affiliations:** 1 State Key Laboratory of Eye Health, Department of Ophthalmology, Shanghai Ninth People’s Hospital, Shanghai Jiao Tong University School of Medicine, Shanghai, 200011 People’s Republic of China; 2 Department of Biological Chemistry and Molecular Pharmacology, Harvard Medical School, Boston, MA USA; 3 Program in Cellular and Molecular Medicine, Boston Children’s Hospital, Boston, MA USA

**Keywords:** Traumatic optic neuropathy, Microglial pyroptosis, Self-amplifying RNA therapy, Disulfiram, Co-delivery LNP, Axon regeneration

## Abstract

**Background:**

Traumatic optic neuropathy (TON) is a devastating cause of irreversible vision loss for which no effective treatment currently exists. Its poor prognosis stems from two major challenges: the limited regenerative capacity of retinal ganglion cells (RGCs) and the hostile, inflammation-driven environment that follows injury.

**Results:**

In this work, using transcriptomic bioinformatic and histopathological analysis, we discovered that mechanical trauma and subsequent neuroinflammation trigger microglial pyroptosis through the NLRP3/CASP1/GSDMD pathway. This process amplifies inflammatory cascades and exacerbates RGC degeneration via microglia-neuron interactions. To overcome these dual barriers, we engineered a microglia-targeted lipid nanoparticle (LNP) platform co-delivering disulfiram (DSF), a selective GSDMD inhibitor, together with self-amplifying mRNA (saRNA) encoding ciliary neurotrophic factor (CNTF). We found that this combinatorial strategy concurrently suppresses pyroptosis-driven neuroinflammation while providing sustained neurotrophic support. Through comprehensive in vitro and in vivo evaluations, the co-delivery system showed enhanced RGC survival, remarkable axonal regeneration, and eventually significant restoration of visual function.

**Conclusions:**

In summary, our results demonstrate that a coordinated strategy targeting both neuroinflammatory mechanisms and regenerative pathways yields superior therapeutic outcomes in TON. This work underscores the potential of integrated RNA-small molecule therapies as a promising multi-target treatment paradigm, with broad applicability for other neuroinflammatory and neurodegenerative diseases.

**Graphical abstract:**

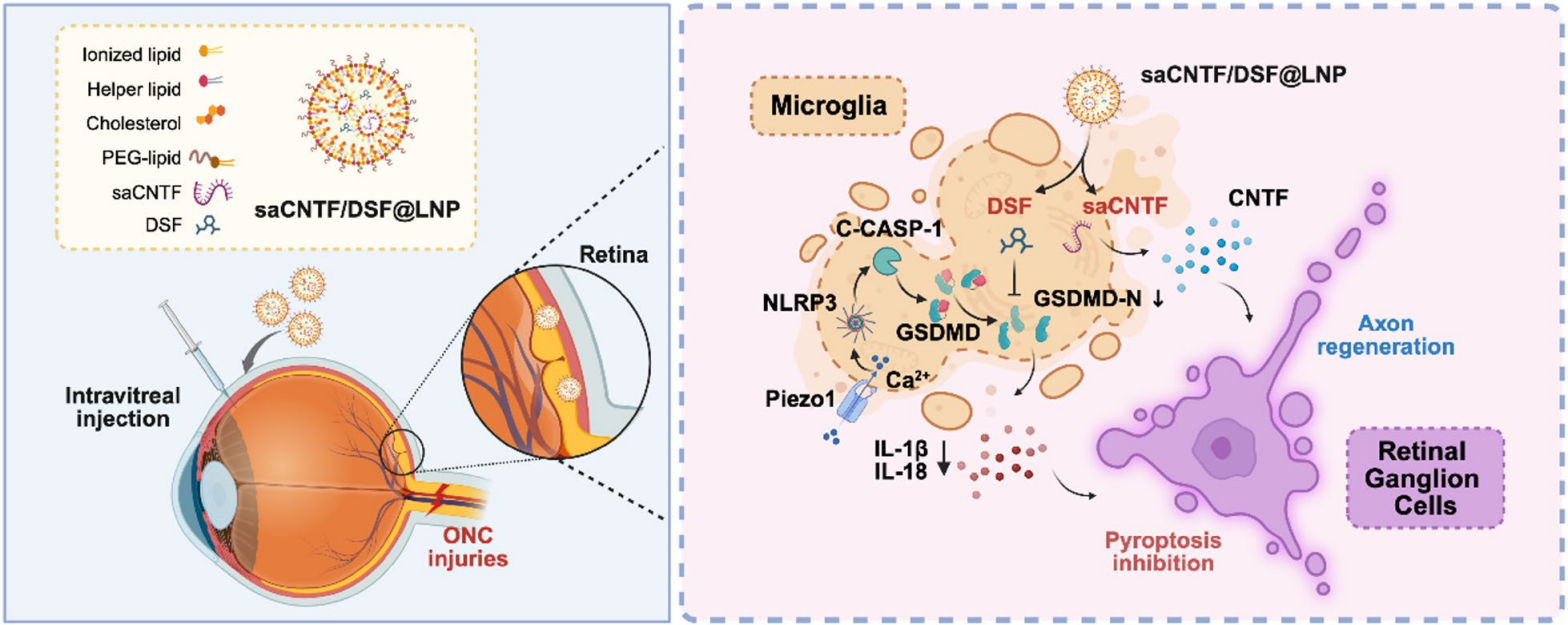

**Supplementary Information:**

The online version contains supplementary material available at 10.1186/s12951-026-04272-x.

## Introduction

Optic nerve injury represents one of the most challenging causes of irreversible blindness worldwide, encompassing a spectrum of conditions such as glaucoma, traumatic optic neuropathy (TON), and other optic neuropathies [1–3]. Central to the pathology of these disorders is the extreme vulnerability of retinal ganglion cells (RGCs), the critical neurons that form the optic nerve by transmitting visual information from the retina to the brain [4]. In adult mammals, RGCs possess limited regenerative capacity [5, 6]. Once injured, they undergo axonal disintegration, somatic loss, and eventual death, leading to permanent visual impairment [7]. Current clinical interventions treating TON, including surgical decompression and high-dose corticosteroids, remain largely palliative, failing to meaningfully promote RGC survival or stimulate functional axon regeneration [8]. Among these disorders, TON often resulting from traffic accidents, falls, or direct cranial impact, stands out as a particularly severe form of nerve injury [9]. It is characterized not only by rapid visual decline but also by intense inflammatory responses that further exacerbate neural damage [10]. The refractoriness of TON to conventional treatments stems from its dual pathogenic nature: the initial mechanical insult and the subsequent neuroinflammatory cascade [11]. Growing evidence indicates that these processes are mechanistically intertwined [12]. Immune cells can sense and respond to mechanical forces, converting physical trauma into biochemical signals that drive inflammation [13, 14].

A pivotal recent study revealed that PIEZO-type mechanosensitive ion channel component 1 (PIEZO1)-mediated mechanotransduction directly activates the NLRP3 inflammasome in a calcium-dependent manner in immune cells, thereby establishing a molecular bridge between mechanical injury and inflammation [15]. Pyroptosis is a highly inflammatory, lytic form of programmed cell death triggered by NLRP3 inflammasome activation, Caspase1 cleavage, and gasdermin D (GSDMD)-dependent pore formation in the cell membrane. These pores facilitate the release of inflammatory cytokines into the local microenvironment, initiating an inflammatory cascade that severely impairs tissue repair [16, 17]. Accumulating evidence has demonstrated elevated levels of pyroptosis-associated proinflammatory cytokines, such as IL-1β, in the context of TON [10, 18]. Nevertheless, whether pyroptosis occurs in TON and can be treated as a therapeutic target remains unclear.

To address this, we performed transcriptomic analysis of retinal tissue in a TON mouse model, with the optic nerve crush (ONC) injury [19], which revealed significant upregulation of pyroptosis-related genes and pathways. Our findings in single-cell RNA sequencing (scRNA-seq) analysis demonstrated that pyroptosis indeed occurs in TON, with microglial cells acting as critical mediators in this process. This mechanism leads to the rupture of microglial cells and the subsequent release of inflammatory mediators into the extracellular milieu. Recent studies have demonstrated that inhibition of NLRP3/CASP1/GSDMD-mediated pyroptosis effectively reversed the inflammatory microenvironment and facilitated the restoration of immune homeostasis, leading to significant inflammation treatment [20] and tissue repair [21]. Notably, the FDA-approved drug disulfiram (DSF) has been repurposed as a potent and selective inhibitor of pyroptosis; it covalently binds to GSDMD and inhibits its pore-forming activity [22], thereby offering a promising therapeutic strategy to suppress acute neuroinflammation. However, the clinical translation of DSF for neurological disorders remains challenging due to its nonselective cellular distribution, narrow therapeutic window, and potential cytotoxicity [23, 24]. These limitations underscore the need for a targeted delivery system to improve both its efficacy and safety profile.

While suppressing pyroptosis may mitigate early microglial activation and inflammatory damage, we assumed that this intervention alone is insufficient to ensure functional recovery. Lasting visual restoration requires not only halting pathological processes but also actively promoting RGC survival and axonal regrowth [25]. To address this need, we turned to neurotrophic factors, among which ciliary neurotrophic factor (CNTF) has emerged as a leading candidate due to its dual capacity to enhance RGC survival and stimulate axon regeneration, particularly for its ability to activate intrinsic growth programs and support synaptic connectivity in the visual system [4]. Despite its promise, its clinical application in recombinant form has been limited by poor stability, short half-life, and inefficient delivery to retinal targets [26].

**Scheme 1:**
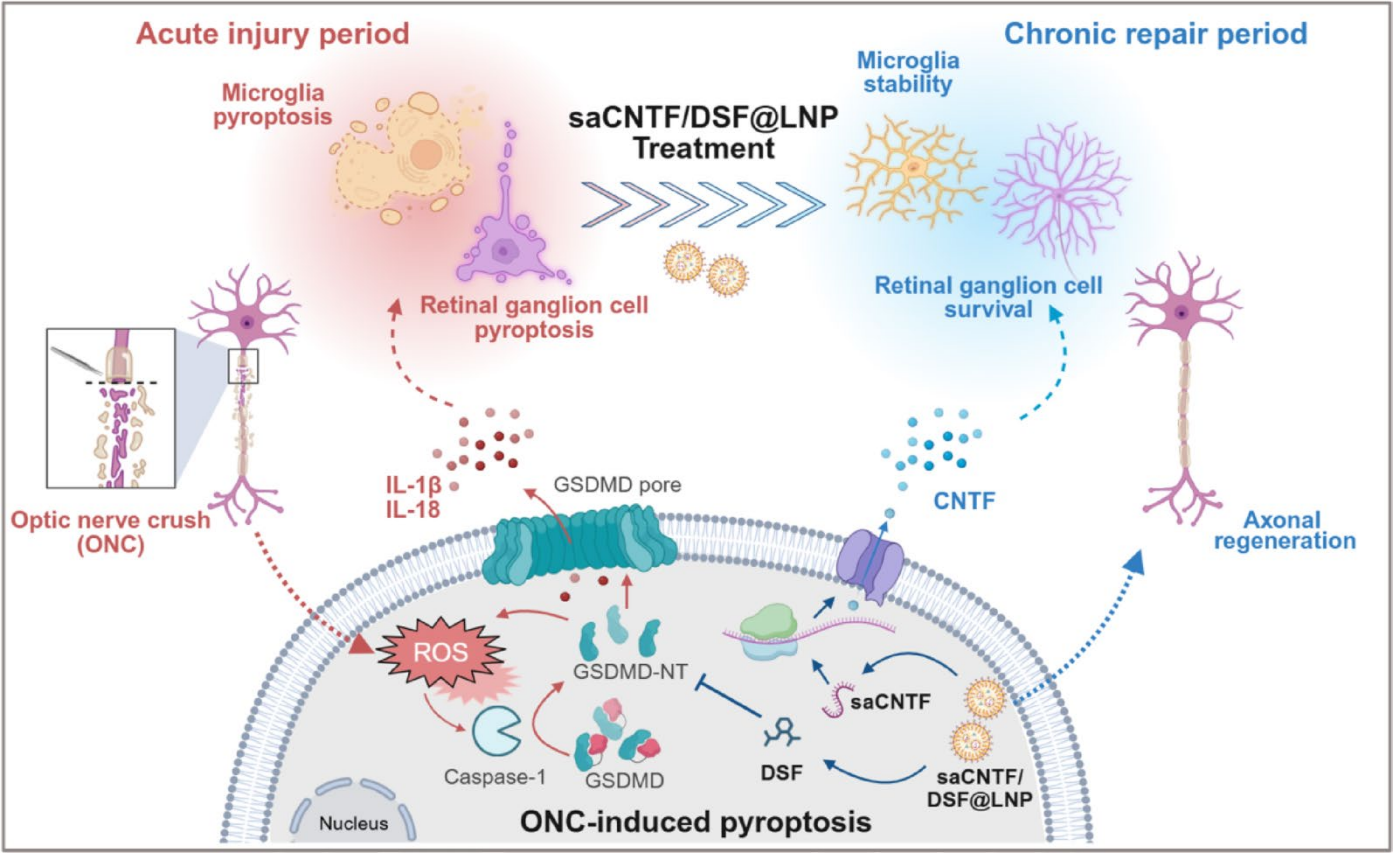
Schematic illustration of the saCNTF/DSF@LNP nano-platform for co-delivering DSF and CNTF saRNA to simultaneously inhibit microglial pyroptosis and provide sustained neurotrophic support, promoting RGC survival and axon regeneration after optic nerve neuropathy

To address these challenges, we developed an integrated nanotherapeutic strategy co-encapsulating DSF and self-amplifying mRNA encoding CNTF (saCNTF) in lipid nanoparticles (saCNTF/DSF@LNP). This combinatory system aims to simultaneously suppress pyroptosis-driven neuroinflammation in the acute phase and provide sustained neurotrophic support for RGC survival and long‐term axon regeneration (Scheme 1). By leveraging saRNA for sustained CNTF expression [27, 28] and LNP for efficient targeted delivery to reduce the off-target toxicity of DSF, we seek to overcome the limitations of conventional protein-based neurotrophic therapies. In this study, the correlation between pyroptosis and the progression of TON was discovered by bioinformatic analyses in the ONC mouse model. We then detailed the design, characterization, and cellular targeting of the saCNTF/DSF@LNP system, demonstrating its efficacy in suppressing microglial pyroptosis, promoting RGC survival, and facilitating axonal regeneration through comprehensive in vitro and in vivo assessments. Finally, the restoration of visual function after saCNTF/DSF@LNP treatment was further demonstrated by animal behavior evaluation.

By synergizing nanomedicine, RNA therapy, and mechanistic immunology, our study provides a novel combinatorial platform for the treatment of optic nerve injuries and presents a translatable strategy for other neuroinflammatory and degenerative diseases.

## Results

### Transcriptomic Profiling Identifies Pyroptosis and Piezo1 Upregulation after ONC

**Fig. 1:**
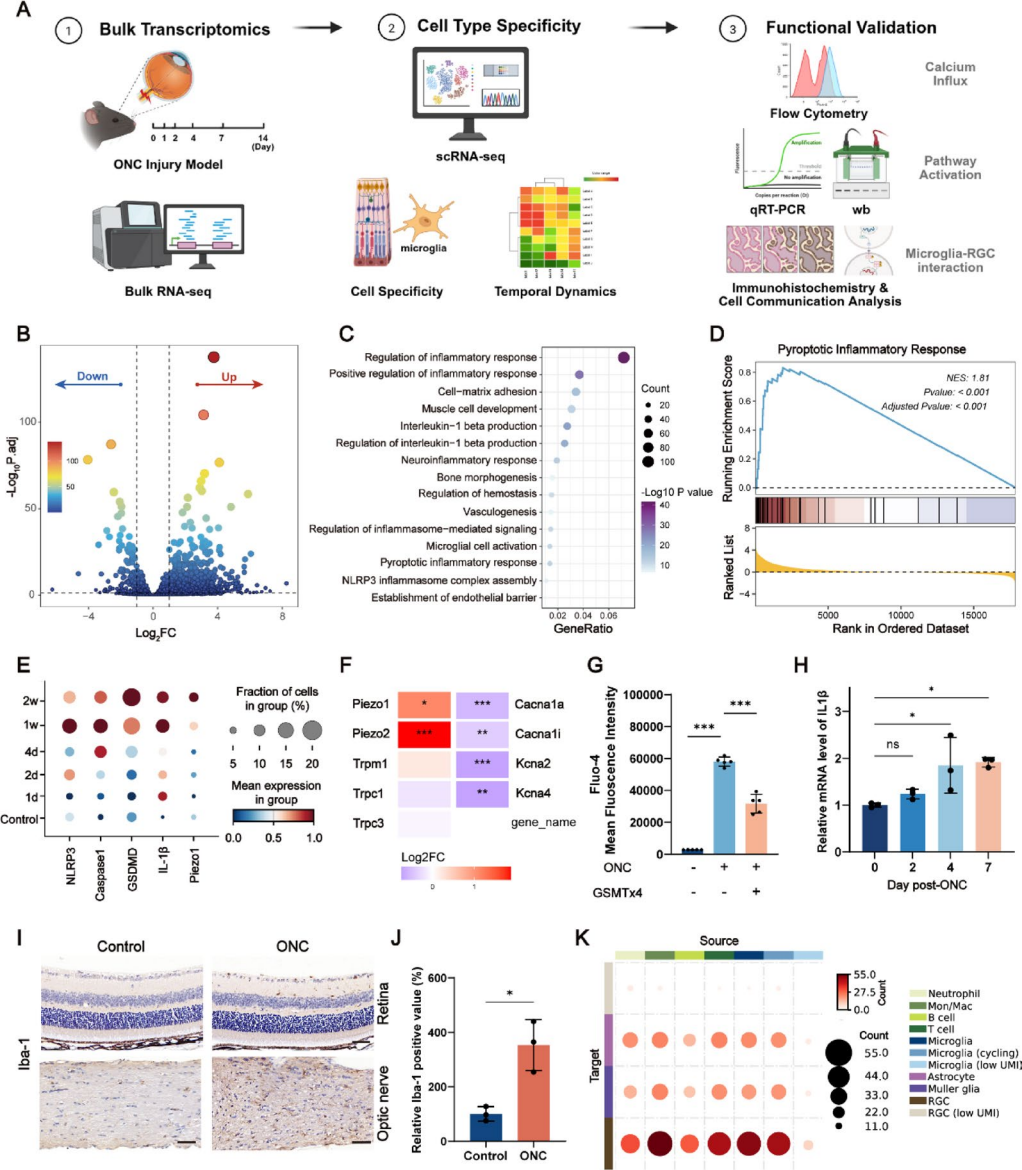
Transcriptomic and functional analyses reveal activation of pyroptosis and Piezo1-mediated calcium signaling in the retina after ONC. (**A**) Schematic overview of the integrated multi-omics and functional validation workflow to investigate pyroptosis activation after optic nerve crush. (**B**) Volcano plot of DEGs between control and ONC retinas at day 7 post-injury. (**C**) GO enrichment analysis of DEGs. (**D**) GSEA enrichment plot for pyroptotic inflammatory response. (**E**) Time-dependent expression of pyroptosis-related genes and Piezo1. (**F**) Heatmap of calcium channel gene expression. (**G**) Representative flow cytometry quantification of Fluo-4 AM mean fluorescence intensity (MFI) in retinal cells from Sham, ONC, and ONC + GsMTx4 (PIEZO1 inhibitor) groups at day 7 post-injury (*n* = 3). (**H**) qRT-PCR analysis of (**H**) NLRP3, (**I**) Caspase1, and (**J**) IL-1β mRNA (*n* = 3). (**K**) Iba-1 immunohistochemical staining in optic nerves at day 7 post-ONC. (**L**) Quantification of Iba-1‑positive area (*n* = 3). Scale bar = 50 μm. (M) Cell interaction network highlighting microglia-RGC crosstalk. Data are mean ± SD, ^*^P < 0.05, ^**^P < 0.01, and ^***^P < 0.001. ns, not significant

To investigate retinal changes following TON, we employed an integrated multi-omics and functional validation workflow (Fig. 1A) to systematically identify and characterize key pathological events. We performed bulk RNA sequencing of mouse retinas 7 days post-ONC. Differential gene expression analysis, conducted using the DESeq2 package, revealed 1,544 significantly dysregulated genes compared to controls, including 1,403 upregulated and 141 downregulated genes (Fig. 1B). Gene Ontology (GO) enrichment highlighted strong associations with inflammatory and neuroinflammatory responses (Fig. 1C). Notably, pyroptosis-related pathways, such as inflammasome-mediated signaling, regulation of inflammasome-mediated signaling, and NLRP3 inflammasome complex assembly, were significantly enriched (Fig. S1). Gene set enrichment analysis (GSEA) further confirmed the upregulation of pyroptosis inflammatory response pathways post-ONC (Fig. 1D and Fig. S2).

Building on these transcriptomic alterations, we next analyzed scRNA-seq data [29] to delineate the temporal dynamics of gene expression following ONC. Our analysis revealed a time-dependent pattern of key pyroptosis-related genes, NLRP3, Caspase1, and IL-1β, showing a sustained increase that peaked approximately one week after ONC (Fig. 1E). Intriguingly, the mechanosensitive ion channel PIEZO1 exhibited a congruent upregulation over time. Notably, among calcium channel genes, only Piezo1 and Piezo2 were significantly elevated after injury (Fig. 1F and Fig. S3). Given the established role of PIEZO1 in transducing mechanical stress into cellular signaling and prior reports linking it to inflammatory cascades, we hypothesized that PIEZO1 might contribute to the activation of the observed pyroptosis pathway following ONC. To test this hypothesis, we first assessed PIEZO1-mediated calcium influx by intravitreal administration of the selective PIEZO1 blocker GsMTx4 upon ONC injury. Flow cytometry analysis using Fluo-4 AM showed that ONC-induced elevated retina calcium influx level was significantly attenuated by GsMTx4 (Fig. 1G), confirming PIEZO1-mediated cation transport following injury.

The induction of pyroptosis was further validated by qRT-PCR, demonstrating a time-dependent upregulation of NLRP3, Caspase1, and IL-1β at day 2, 4, and 7 post-ONC (Fig. 1H and Fig. S4). While elevated at all post-injury time points, the expression levels of these genes did not show statistically significant further increases between days 2, 4, and 7, indicating a rapid and sustained activation of the pathway. Critically, western blot analysis confirmed the presence of the cleaved, active form of GSDMD (GSDMD-NT) in retinal lysates at days 2, 4, and 7 following ONC, providing direct protein-level evidence of pyroptosis executor activation in vivo (Fig. S5). Elevated IL-1β was also detected in optic nerve sections (Fig. S6). Furthermore, a marked increase in reactive oxygen species (ROS) was observed and spatially localized within the ONC retina (Fig. S7), supporting the presence of oxidative stress-mediated pyroptosis activation and inflammatory microenvironment formation [30].

To identify retinal cell types involved in pyroptosis, we analyzed scRNA-seq data, which revealed that pyroptosis-related genes were predominantly expressed in microglia (Fig. S8 and Fig. S9), suggesting a central role of microglial pyroptosis in ONC-induced retinal damage. Concurrently, immunohistochemistry of the optic nerve performed at Day 7 post-ONC showed elevated ionized calcium-binding adapter molecule 1 (Iba-1) expression and an increased number of microglia following ONC (Fig. 1I and J). We next investigated intercellular crosstalk among immune cells, neurons, and glial cells, which identified robust interactions between microglia and RGCs (Fig. 1K). This specific microglia-RGC interaction was corroborated by comprehensive cell communication analysis across all major retinal cell types (Fig. S10).

In summary, our transcriptomic data and functional experiments implicated the pyroptosis pathway and PIEZO1 upregulation following ONC, which may influence RGC fate after injury.

### Preparation and Characterization of saCNTF/DSF@LNP

**Fig. 2:**
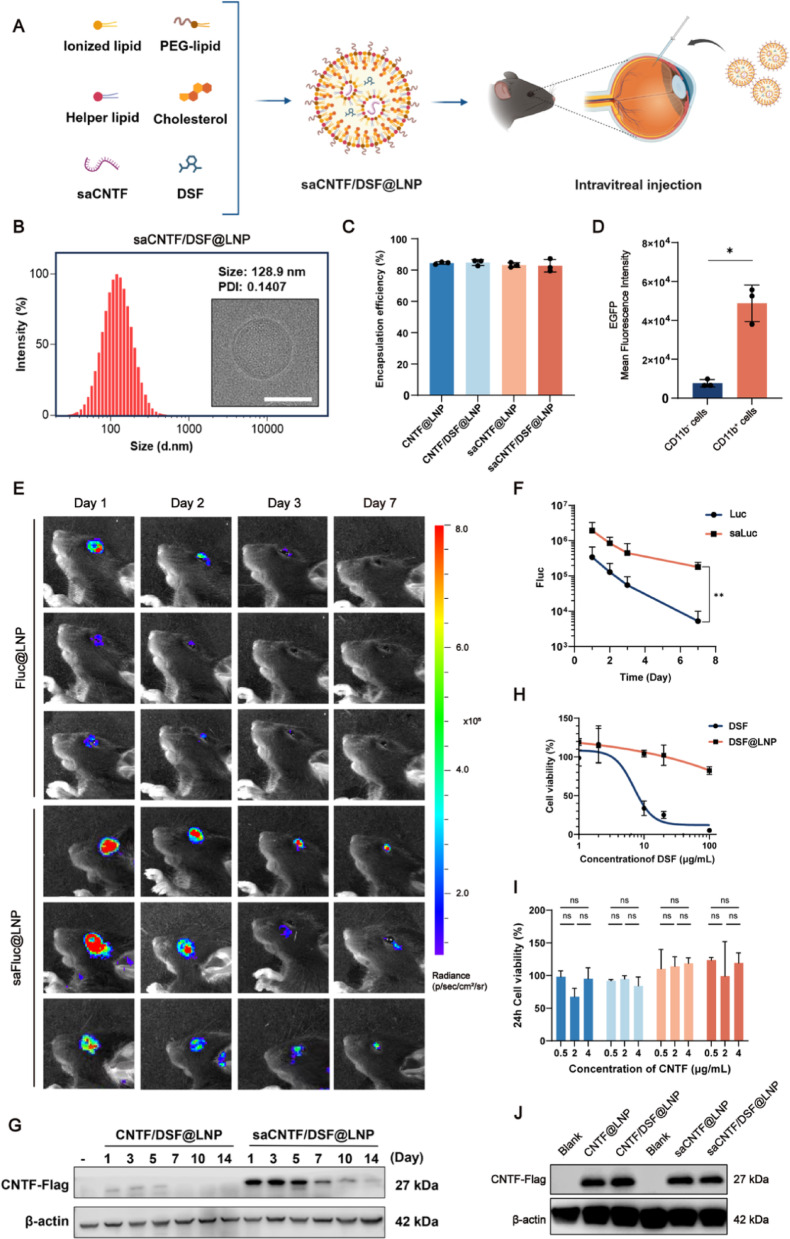
Preparation and characterization of saCNTF/DSF@LNP. (**A**) Schematic design of saCNTF/DSF@LNP composition. The graphic was created with BioRender.com. (**B**) Size distribution and Cryo-EM imaging of saCNTF/DSF@LNP. Scale bar = 100 nm. (**C**) RNA encapsulation efficiency of CNTF@LNP, CNTF/DSF@LNP, saCNTF@LNP, and saCNTF/DSF@LNP, as measured by RiboGreen assay (*n* = 3). (**D**) Flow cytometry analysis of EGFP expression in CD11b^+^ and CD11b^-^ retinal cells from ONC mice treated with EGFP mRNA-loaded LNPs (*n* = 3). (**E**) In vivo bioluminescence imaging of mice intravitreally injected with LNP encapsulating firefly-luciferase mRNA or saRNA. (**F**) Quantification of bioluminescence intensity over time (*n* = 3). (**G**) Western blot analysis of CNTF protein levels in retinal lysates at days 1, 3, 5, 7, 10, and 14 after a single intravitreal injection of CNTF/DSF@LNP or saCNTF/DSF@LNP. (**H**) Cell viability (CCK-8 assay) of BV2 microglia treated for 24 h with free DSF or DSF@LNP across a range of concentrations (*n* = 5). (**I**) Cell viability of BV2 cells treated with CNTF@LNP, CNTF/DSF@LNP, saCNTF@LNP, or saCNTF/DSF@LNP (0.5–4 µg/mL, CNTF-equivalent) for 24 h. (**J**) Western blot analysis of CNTF protein expression in BV2 cells at 48 h post-transfection with CNTF@LNP, CNTF/DSF@LNP, saCNTF@LNP, or saCNTF/DSF@LNP. Data are mean ± SD, ^*^P < 0.05, ^**^P < 0.01, and ^***^P < 0.001. ns, not significant

To address both RGC regeneration and microglial pyroptosis-mediated inflammatory cascade post-TON, we prepared LNPs co-encapsulated with saCNTF and DSF (saCNTF/DSF@LNP) for TON treatment via intravitreal injection by using nanoprecipitation method based on vortex mixing (Fig. 2A) [31]. The particle size and polydispersity index (PDI) of saCNTF/DSF@LNP were 128.9 nm and 0.14, respectively, characterized by dynamic light scattering (DLS) analysis and Cryo-EM imaging (Fig. 2B). We also prepared LNPs co-encapsulated with CNTF mRNA and DSF (CNTF/DSF@LNP), LNPs encapsulated only with CNTF mRNA (CNTF@LNP), or saCNTF (saCNTF@LNP), and DSF-loaded LNPs (DSF@LNP) for controls. Their particle size and PDI were shown in Figures S11 and S12. The RNA encapsulation efficiency of all LNPs was over 80% as measured by RiboGreen assay (Fig. 2C).

We first wanted to assess the in vivo cell targeting of the LNP delivery system after intravitreal injection, as the FDA-approved formulation has been commercialized only for intravenous and intramuscular administration [32, 33]. EGFP reporter mRNA-loaded LNPs were prepared and intravitreally injected into ONC-injured mice, and retinal cells were collected for flow cytometry analysis. Surprisingly, we found that the EGFP expressing efficiency in CD11b^+^ microglia was approximately 6.3-fold higher than that in CD11b^−^ cells (Fig. 2D and Fig. S13). To further investigate the mechanism of CD11b^+^ cell-specific targeting, we performed an in vitro phagocytosis inhibition assay by pretreating BV2 cells with scavenger receptor B1 (SR-B1) blocker BLT-1 follow EGFP@LNP transfection. The results showed SR-B1 pretreatment significantly reduced EGFP expression efficiency by approximately 50% (Fig. S14), indicating scavenger receptor-involved microglial phagocytosis.

We then compared the in vivo expression levels of mRNA and saRNA by using firefly-luciferase reporter mRNA (Fluc)- and saRNA (saFluc)-loaded LNPs. SaFluc@LNP showed enhanced expression efficiency and prolonged expression duration for up to 7 days compared with Fluc@LNP after intravitreal injection (Fig. 2E and F). To further investigate the in vivo protein expression of saCNTF/DSF@LNP in comparison to CNTF/DSF@LNP, we assessed CNTF levels by western blotting using retinal tissue samples following a single intravitreal injection of either CNTF/DSF@LNP or saCNTF/DSF@LNP at indicated time points. CNTF protein level from CNTF/DSF@LNP group was low and only detectable from day 1 to day 3, and declined rapidly thereafter. In contrast, expression from the saCNTF/DSF@LNP remained robust at Day 7 and Day 10, and persisted at measurable levels for up to 14 days (Fig. 2G). This result confirms the enhanced and prolonged expression profile of the saRNA platform for neurotrophic factor delivery. Similar results were obtained in the in vitro transfection assay with Flag-tagged CNTF@LNP and saCNTF@LNP (Fig. S15). Furthermore, analysis of endogenous CNTF expression at Day 4 post-ONC revealed no significant upregulation compared to uninjured retinas (Fig. S16), highlighting a deficient intrinsic neurotrophic response and justifying our therapeutic supplementation approach.

DSF, an FDA-approved drug for antialcoholism and antitumor treatment [34], has been reported to antagonize GSDMD-mediated pyroptosis but with potent cytotoxicity [22], limiting its clinical application for pyroptosis inhibition. To address this issue, we loaded DSF into LNPs for sustained intracellular release. Cell viability assays in BV2 microglia showed that DSF@LNP significantly reduced cytotoxicity and broadened the therapeutic window compared to free DSF at both 24 and 48 h post-treatment. While free DSF exhibited severe toxicity at concentrations above 2 µg/mL, DSF@LNP maintained markedly higher cell viability across the tested range (1–20 µg/mL). The 48 h IC_50_ values of free DSF and DSF@LNP were measured as 6.9 µg/mL and 28.7 µg/mL, respectively. (Fig. 2H and Fig. S17).

To ensure the biocompatibility of all formulations used in this study, we next assessed the cell viability of BV2 cells treated with CNTF@LNP, CNTF/DSF@LNP, saCNTF@LNP, and saCNTF/DSF@LNP. All LNP formulations demonstrated excellent safety profiles, maintaining high cell viability across a range of therapeutically relevant concentrations (0.5–4 µg/mL, CNTF-equivalent) after 24- and 48-h exposures (Fig. 2I and Fig. S18). This confirms that neither the CNTF/saCNTF payload nor its co-encapsulation with DSF introduces significant cytotoxicity. Next, the expression efficiency of CNTF@LNP, CNTF/DSF@LNP, saCNTF@LNP, and saCNTF/DSF@LNP was tested in BV2 cells after 48 h post-transfection, showing that DSF incorporation did not influence the expression level of loaded CNTF or saCNTF (Fig. 2J).

### saCNTF/DSF@LNP Mediates In Vitro Inhibition of Pyroptosis and Pyroptosis Propagation

**Fig. 3:**
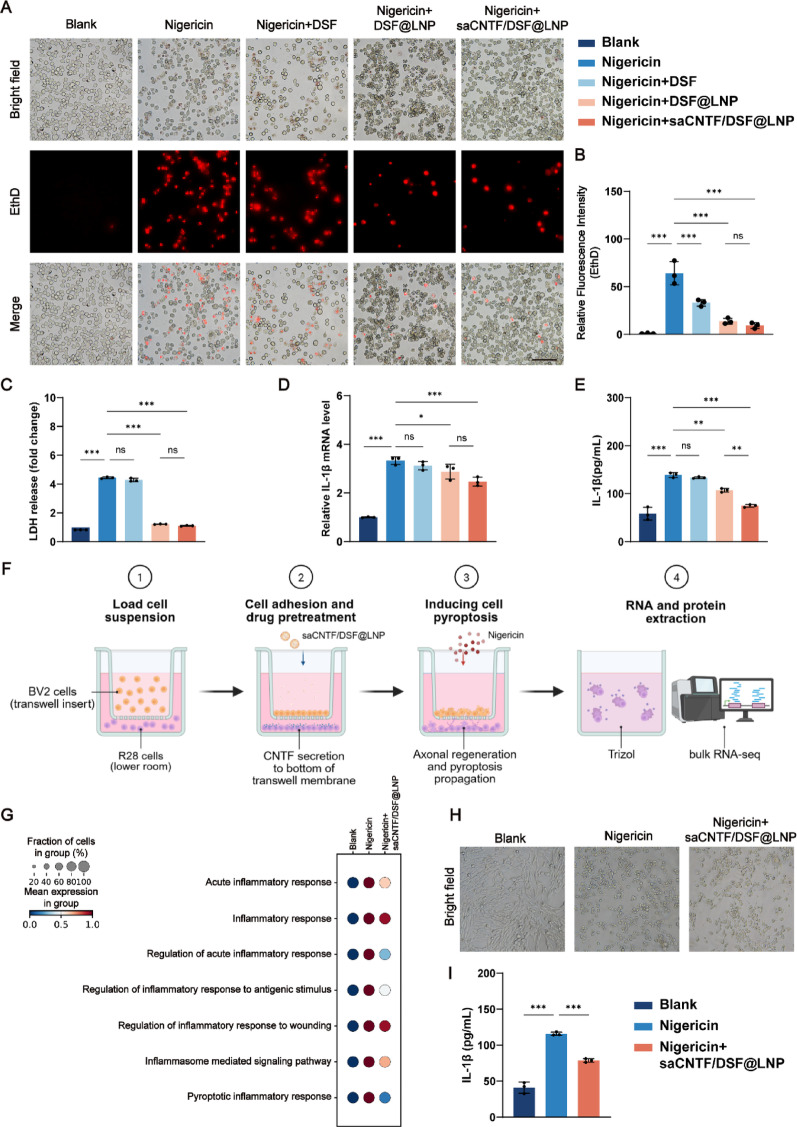
SaCNTF/DSF@LNP inhibits pyroptosis in microglia and attenuates inflammatory injury in retinal neurons in vitro. (**A**) Representative images showing pyroptotic morphology and EthD staining in BV2 cells. Cells were pretreated with the indicated LNPs for 12 h, followed by nigericin (20 µm) stimulation for 6 h. Scale bars = 100 μm. (**B**) Cell death rate and (**C**) LDH release in BV2 cells across treatment groups (*n* = 3). (**D**) qRT-PCR analysis of IL-1β mRNA expression in BV2 cells (*n* = 3). (**E**) ELISA of IL-1β levels in cell culture supernatants (*n* = 3). (**F**) Schematic of the transwell co-culture system with BV2 (upper chamber) and R28 cells (lower chamber). (**G**) Dot plot illustrating the enrichment scores of GO terms across the three groups of R28 cells after 24 h of co-culture. (**H**) Morphology of R28 cells after 24 h of co-culture under pyroptotic stimulation. Scale bar = 100 μm. (**I**) IL-1β secretion in R28 culture supernatant measured by ELISA after 24 h of co-culture (*n* = 3). Data are mean ± SD, ^*^P < 0.05, ^**^P < 0.01, and ^***^P < 0.001. ns, not significant

Given that GSDMD-mediated pyroptosis was strongly correlated with retinal and optic nerve inflammation in ONC mice, we further tested the in vitro pyroptosis inhibition of saCNTF/DSF@LNP co-delivery system. Nigericin, a well-established agonist of NLRP3-activated pyroptosis [35], was used to induce pyroptosis in BV2 cells. We observed that free DSF conferred only partial protection, presumably limited by its inherent cytotoxicity. In contrast, both DSF@LNP and saCNTF/DSF@LNP significantly and equivalently enhanced cell survival, as evidenced by reduced EthD staining (Fig. 3A and B) and decreased LDH release (Fig. 3C).

qRT-PCR analysis revealed that nigericin stimulation significantly upregulated NLRP3, Caspase1, and IL-1β expression by 1.5–3.5-fold. which were markedly and to a comparable extent restored by saCNTF/DSF@LNP treatment (Fig. 3D and Fig. S19). In contrast, GSDMD expression remained unchanged (Fig. S19), indicating that saCNTF/DSF@LNP specifically inhibits the upstream NLRP3 inflammasome pathway. Furthermore, ELISA revealed that the secretion level of IL-1β, a hallmark proinflammatory cytokine in pyroptosis, was significantly increased upon nigericin stimulation, and was significantly reduced by both LNP treatments. A slight but statistically greater reduction was observed in the saCNTF/DSF@LNP group compared to DSF@LNP (Fig. 3E), suggesting a possible additional immunomodulatory effect of CNTF. Similar results were obtained in immunoblotting analysis, where the upregulation of cleaved IL-1β expression induced by nigericin was substantially attenuated by saCNTF/DSF@LNP (Figure S20), confirming the in vitro pyroptosis suppression of saCNTF/DSF@LNP. To be note, the pyroptosis inbition of DSF@LNP and saCNTF/DSF@LNP were almost comparable, presumably because the primary suppression of NLRP3 inflammasome activation and IL-1β maturation in this acute model is mainly attributed to the disulfiram (DSF) component, which is formulated by the same methodology.

Since GSDMD-mediated pyroptosis was reported to intercellularly propagate to bystander cells [36], we hypothesized that microglial pyroptosis might also trigger the nearby RGCs’ death and amplify inflammatory cascades, which can be further inhibited by saCNTF/DSF@LNP. To test this, we established a transwell-based co-culture system with BV2 as effector cells in the upper chamber and R28, a retinal precursor cell line with biological properties similar to RGCs, as target cells in the lower chamber (Fig. 3F). BV2 cells pretreated with or without saCNTF/DSF@LNP were then treated with nigericin, and R28 cells were assessed for bulk RNA sequencing analysis and pyroptotic changes. The transcriptomic sequencing further confirmed the significant upregulation of multiple inflammation-associated signaling pathways mediated by nigericin stimulation, which was further restored by saCNTF/DSF@LNP (Fig. 3G). Microscopy revealed stark morphological differences: control R28 cells displayed intact neuronal processes, whereas nigericin-treated cells showed severe pyroptosis, including axonal loss, somatic swelling, and bubble-like protrusions. saCNTF/DSF@LNP treatment markedly attenuated these features, preserving axons and reducing swelling and membrane bubbles (Fig. 3H). Consistent with morphology, ELISA indicated that nigericin stimulation increased IL-1β secretion nearly 3-fold (to ∼110 pg/mL), which saCNTF/DSF@LNP significantly reduced to ∼80 pg/mL (Fig. 3I), confirming suppression of pyroptosis-induced inflammation.

Taken together, our in vitro results demonstrated that saCNTF/DSF@LNP effectively inhibits GSDMD-mediated pyroptosis in BV2 microglia, attenuates inflammatory cytokine release, and confers morphological protection to co-cultured R28 cells. These findings highlight the potential of saCNTF/DSF@LNP as a promising therapeutic strategy for countering microglia-driven neuroinflammation.

### saCNTF/DSF@LNP Inhibits In Vivo Pyroptosis and Inflammation under ONC

**Fig. 4:**
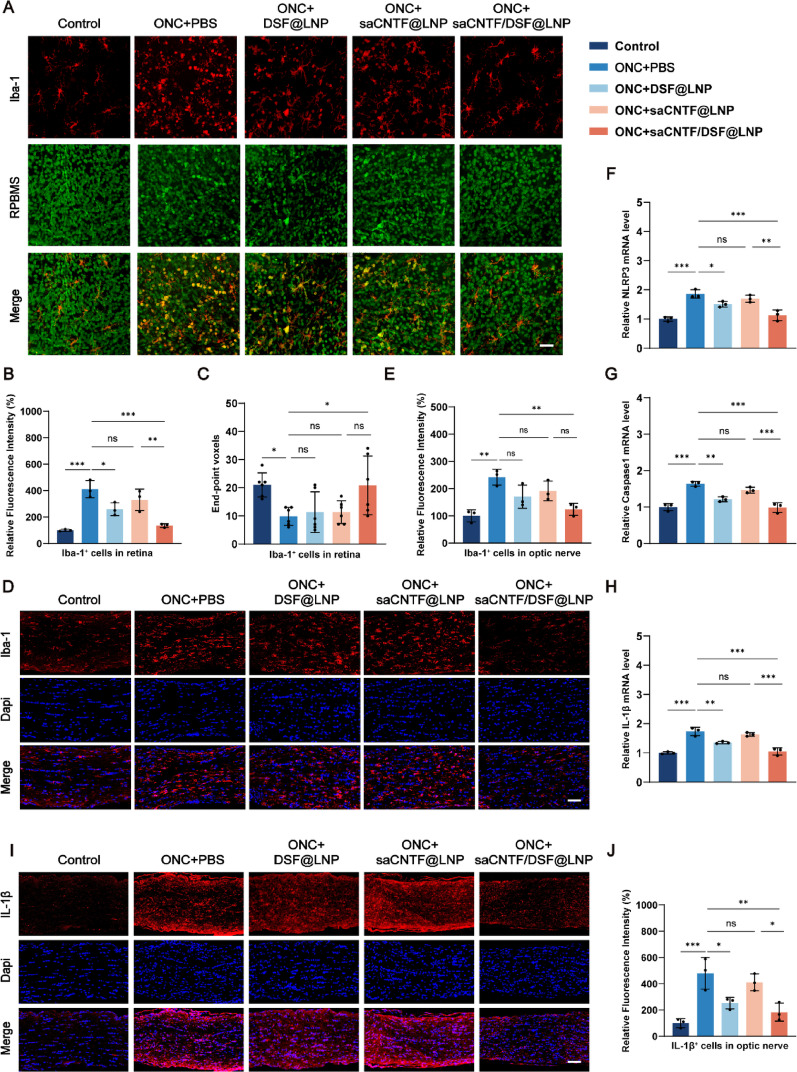
saCNTF/DSF@LNP suppresses microglial activation and pyroptosis-related inflammation in the retina and optic nerve post-ONC. (**A**) Representative immunofluorescence images of retinal microglia (Iba-1, red) and RGCs (RBPMS, green) 4 days post-ONC under the indicated treatments. Scale bar = 20 μm. (**B**) Quantification of Iba-1 mean fluorescence intensity in retinal microglia (*n* = 3). (**C**) Quantitative analysis of the number of endpoints per microglial cell across groups (*n* = 6). (**D**) Iba-1 immunofluorescence in optic nerve sections. Scale bar = 100 μm. (**E**) Quantification of Iba-1 intensity in optic nerve tissue at 7 days post-ONC. qRT-PCR analysis of (**F**) NLRP3, (**G**) Caspase1, and (**H**) IL-1β mRNA expression in retinal tissue at 4 days post-ONC (*n* = 3). (**I**) IL-1β immunofluorescence in optic nerve sections. Scale bar = 100 μm. (**J**) Quantification of IL-1β intensity in optic nerve tissue at 4 days post-ONC (*n* = 3). Data are mean ± SD, ^*^P < 0.05, ^**^P < 0.01, and ^***^P < 0.001. ns, not significant

Under pathological conditions, activated microglia not only shift from a ramified to an amoeboid morphology and proliferate but also migrate to the ganglion cell layer (GCL), gaining the capacity to inflict RGC damage by releasing pro-inflammatory mediators including TNF-α, interleukins, and NO [37, 38]. Retina exposed to external injuries may also cause microglia overactivation, leading to subsequent inflammasome assembly and pyroptosis [39]. Thus, we observed the morphological and quantitative changes of retinal microglia after ONC and their positional relationship with RGC through immunofluorescence staining of Iba-1, a microglia marker, and RPBMS, an RGC marker. Under ONC injury, we found that the number of microglia increased, the branch length decreased, and the amoeboid morphology increased, as previously reported. The saCNTF/DSF@LNP treatment group showed restored microglial activation by decreased cell number, increased length, and number of microglial cell branches, compared with DSF@LNP and saCNTF@LNP treatment groups (Fig. 4A-C, Fig. S21). Iba-1^+^ microglia were also stained in optic nerve tissue. ONC injury showed enhanced microglia infiltration, which was restored by saCNTF/DSF@LNP treatment (Fig. 4D and E).

Moreover, to investigate the in vivo pyroptosis inhibition in RGCs upon saCNTF/DSF@LNP treatment, pyroptosis-related molecules were further detected. The qRT-PCR results showed that the expression of NLRP3, Caspase1, and IL-1β in the retina was significantly elevated after ONC injury, and recovered by DSF@LNP and saCNTF/DSF@LNP treatment but not saCNTF@LNP (Fig. 4F-H). Similar results were obtained by Caspase1 staining in the retinal ganglion cell layer (GCL) (Fig. S22) and by IL-1β staining in the optic nerve tissues (Fig. 4I and J), respectively.

Consequently, these results indicate the inhibition of microglial activation and pyroptosis via LNP-encapsulated DSF, and more importantly, the attenuated inflammatory microenvironment in both retina and optic nerve by saCNTF/DSF@LNP treatment.

### saCNTF/DSF@LNP Provides Sustained Post-injury Protection of RGCs

**Fig. 5:**
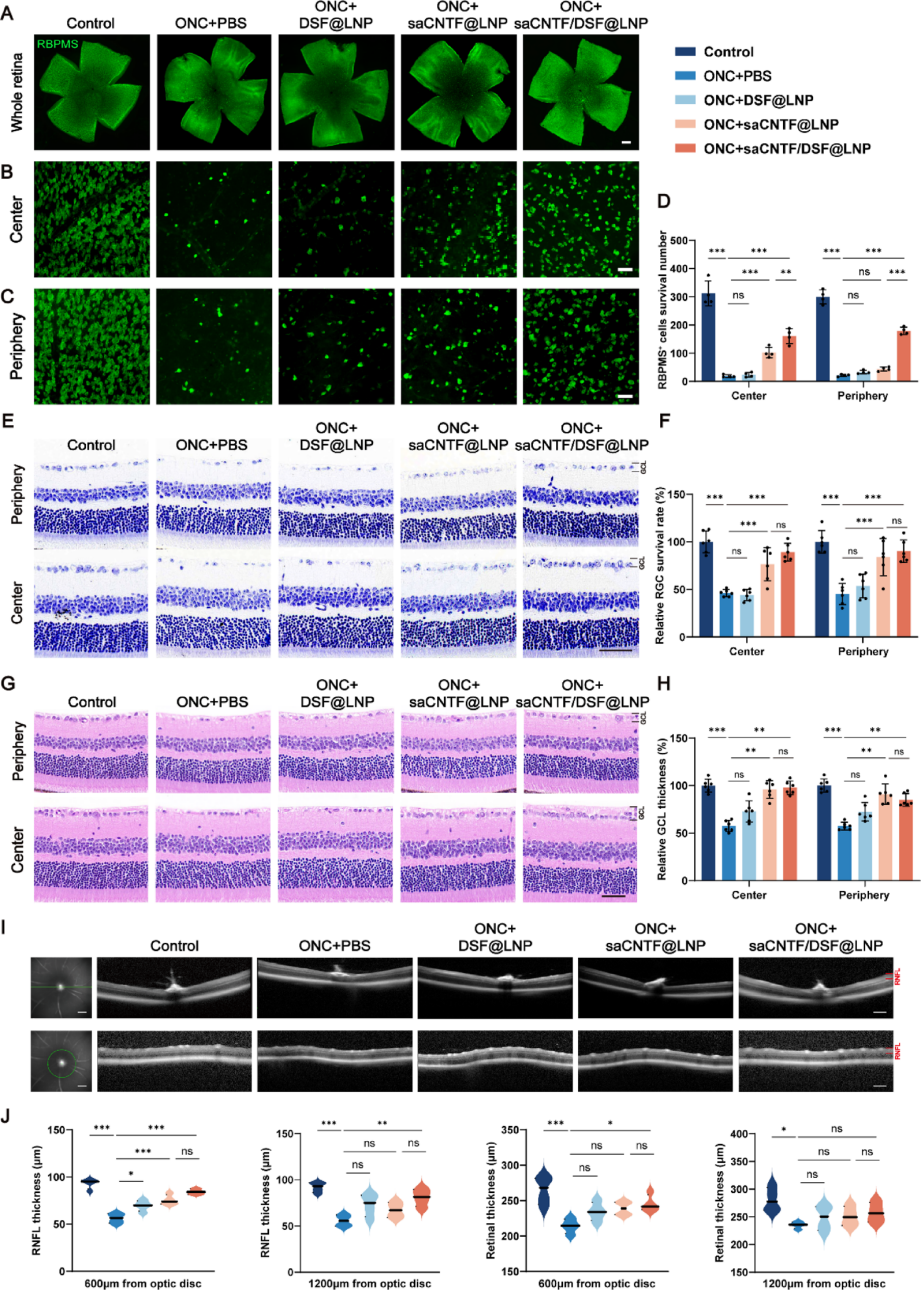
SaCNTF/DSF@LNP treatment attenuates RGC loss and preserves retinal structure after ONC. (**A**) Representative images of whole-mounted retinas immunostained for RBPMS (RGCs) 14 days post-ONC. Scale bar = 200 μm. (**B**, **C**) Higher-magnification views of RGCs in central and peripheral retina. Scale bar = 20 μm. (**D**) Quantification of RGC density (*n* = 4). (**E**) Nissl staining of retinal sections at 14 days post-ONC. The boxed region indicates the GCL area used for counting. Scale bar = 50 μm. (**F**) Quantification of RGC density via Nissl staining (*n* = 6). (**G**) H&E staining of retinal sections at 14 days post-ONC. GCL thickness was measured at 300 μm from the optic nerve head and retinal margin. Scale bar = 50 μm. (**H**) Quantification of GCL thickness (*n* = 6). (**I**) Representative OCT images of retinas pre- and 4 weeks post-ONC and treatments. Scale bar = 200 μm. (**J**) Retinal thickness and RNFL thickness measured at 600 μm and 1200 μm from the optic disc (*n* = 4). Data are mean ± SD, ^*^P < 0.05, ^**^P < 0.01, and ^***^P < 0.001. ns, Not significant

ONC injury triggers acute neuroinflammation and rapid RGC degeneration, necessitating timely intervention [40]. The slower secondary degeneration phase offers a critical therapeutic window for saRNA-based treatment [41, 42]. Therefore, we administered intravitreal injections of various LNPs immediately following ONC. To first quantify the neuroprotective effect of saCNTF/DSF@LNP, we assessed RGC survival by immunostaining at 4 weeks post-injury. saCNTF/DSF@LNP treatment increased RGC survival by approximately 45% compared to the ONC group, with survival rates also surpassing those in DSF@LNP and saCNTF@LNP controls by about 20% and 10%, respectively (Fig. 5A-D), collectively demonstrating the potent neuroprotective efficacy of saCNTF/DSF@LNP.

To further validate RGC survival using the histological method, we performed Nissl staining at 2 weeks (Fig. 5E). This analysis confirmed that saCNTF@LNP and saCNTF/DSF@LNP significantly restored RGC counts relative to the ONC group. In Fig. 5F, results showed that both saCNTF@LNP and saCNTF/DSF@LNP significantly restored RGC counts compared to the ONC group. Specifically, relative to the ONC group (central: 46%, peripheral: 45%), saCNTF@LNP increased RGC survival by approximately 30% centrally and 39% peripherally, while saCNTF/DSF@LNP enhanced RGC survival by approximately 43% centrally and 45% peripherally. In contrast, DSF@LNP, which lacks functional CNTF expression, did not show a significant protective effect. These findings further confirm the specific role of CNTF in promoting RGC survival.

We next evaluated whether RGC preservation translated into the maintenance of retinal layer architecture by assessing GCL thickness via H&E staining. Measurements taken 300 μm from the optic nerve head and retinal margin (Fig. 5G) revealed that saCNTF/DSF@LNP markedly preserved GCL morphology, increasing thickness by 40% centrally and 28% peripherally compared to ONC controls (Fig. 5H), indicating substantial structural protection at the tissue level.

Finally, to non-invasively monitor retinal integrity over time, we employed optical coherence tomography (OCT). Longitudinal scans confirmed pronounced retinal thinning post-ONC, especially 600 μm from the optic disc. After 4 weeks, retinal thickness was significantly reduced at both 600 μm and 1200 μm in ONC mice, while saCNTF/DSF@LNP treatment effectively mitigated this thinning, showing no statistically significant difference from baseline at either location. Similarly, retinal nerve fiber layer (RNFL) thickness was preserved in saCNTF/DSF@LNP-treated animals, in contrast to significant reductions in the ONC group (Fig. 5I and J).

Collectively, these in vivo results demonstrate that saCNTF/DSF@LNP effectively preserves the structural integrity of the RGC layer by promoting neuronal survival and specifically curbing its pathological thinning after injury.

### saCNTF/DSF@LNP Promotes Robust RGC Axonal Protection and Regeneration

**Fig. 6:**
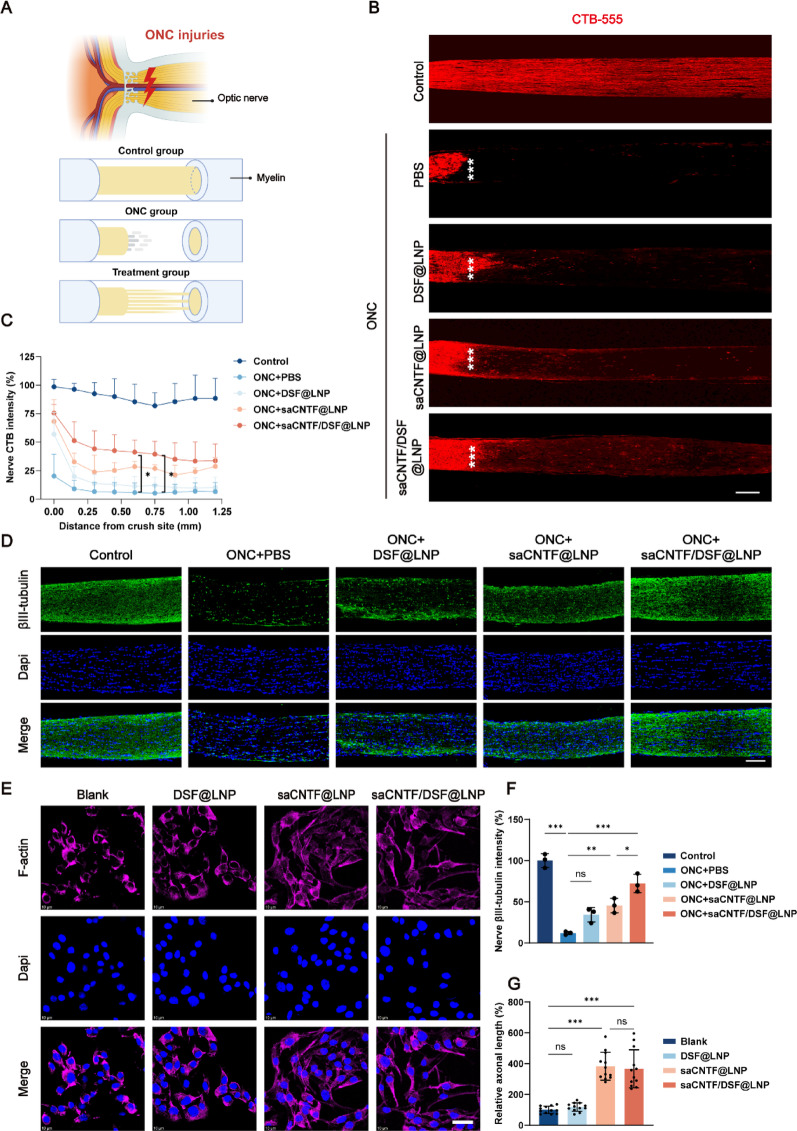
saCNTF/DSF@LNP promotes RGC axon regeneration after ONC injury. (**A**) Schematic of axonal integrity and regeneration in the optic nerve under normal, ONC-injured, and the indicated treatment groups. (**B**) Representative images of CTB^+^ RGC axons in optic nerves from uninjured, ONC (PBS-treated), and LNP-treated mice at 28 days post-injury. ***Indicating crush side. Scale bar = 100 μm. (**C**) Quantification of CTB fluorescence intensity (left) and number of regenerating axons with respect to distance from the lesion site (right) (*n* = 3). (**D**) Representative images of βIII-tubulin^+^ axons in optic nerve sections at 28 days post-ONC. Scale bar = 100 μm. (**E**) Quantification of βIII-tubulin^+^ axon regrowth (*n* = 3). (**F**) Phalloidin staining of the cytoskeleton in R28 cells after 24 h of treatment. Scale bar = 20 μm. (**G**) Quantification of axonal length in R28 cells (*n* = 12). Data are mean ± SD, ^*^P < 0.05, ^**^P < 0.01, and ^***^P < 0.001. ns, Not significant

Axonal regeneration is essential for functional recovery after optic nerve injury but remains limited in the adult mammalian CNS [4]. While CNTF has been established to enhance RGC survival and axonal regrowth [43], its targeted delivery is crucial for efficacy. A schematic of the ONC model and treatment strategy is provided in Fig. 6A, illustrating the injury site posterior to the eyeball, where axonal integrity and transport are disrupted. Whereas ONC resulted in reduced axonal density and disorganized regrowth proximal to the lesion, saCNTF/DSF@LNP treatment supported extensive regeneration beyond the injury site toward the optic chiasm.

As illustrated in the ONC model schematic (Fig. 6A), the crush injury disrupts axonal integrity and transport, creating a challenging environment for regeneration. Consistent with this, ONC resulted in reduced axonal density and disorganized regrowth near the lesion, whereas saCNTF/DSF@LNP treatment supported extensive regeneration beyond the injury site toward the optic chiasm.

To quantitatively evaluate axonal regeneration, we performed anterograde tracing using intravitreal injection of CTB-555 (Fig. 6B). At 28 days post-ONC, CTB signal intensity in the optic nerve was reduced to ∼6% of uninjured controls. In contrast, saCNTF/DSF@LNP treatment restored CTB labeling to approximately 42% (Fig. 6C). Furthermore, saCNTF/DSF@LNP significantly increased the number of axons regenerating beyond 600 μm distal to the crush site, indicating robust recovery of axonal transport and long-distance regrowth.

We next assessed axonal structural integrity and regenerative activity using immunofluorescence markers. Analysis revealed a pronounced decrease in βIII-tubulin expression after ONC, indicative of axonal degeneration, which was markedly preserved by saCNTF/DSF@LNP treatment (Fig. 6D and F). To specifically identify regenerating axons, we performed GAP43 co-staining with CTB tracing. At day 28, CTB⁺ axons beyond the lesion in saCNTF/DSF@LNP-treated mice were consistently GAP43⁺ (Fig. S23), confirming that these axons were actively growing and not merely spared from degeneration.

To further validate the regenerative effect of saCNTF/DSF@LNP in a controlled setting, we turned to an in vitro model using R28 retinal cells. Based on the Bulk RNA sequencing, we found that the DEGs between the saCNTF/DSF@LNP and Nigericin groups were significantly enriched in various GO terms, including the regulation of cell growth, axon guidance, and nerve development (Fig. S24). Notably, pathways related to axon regeneration, such as the Wnt signaling pathway and the mTOR signaling pathway, showed strong enrichment as well (Fig. S25). Cytoskeletal staining showed that both saCNTF/DSF@LNP and saCNTF@LNP significantly enhanced axonal outgrowth after 24 h compared to blank and DSF@LNP controls (*p* < 0.001; Fig. 6E and G), underscoring the essential role of CNTF in driving axon elongation independently of the in vivo microenvironment. Since saCNTF@LNP and saCNTF/DSF@LNP have the comparable in vitro expression level (Fig. 2J), it is expected that they both showed strong neurite outgrowth with no significant differences observed.

In summary, these results demonstrate that saCNTF/DSF@LNP effectively protects RGC axons from degeneration and stimulates robust, long-distance regeneration in vivo, further supported by enhanced axonal growth in vitro. The ability of saCNTF/DSF@LNP to promote both structural and molecular correlates of regeneration highlights its significant therapeutic potential for optic nerve injuries and other CNS disorders.

### saCNTF/DSF@LNP Mediates Comprehensive Functional Visual Recovery

Functional recovery of vision is the ultimate indicator of successful intervention following optic nerve damage. To determine whether saCNTF/DSF@LNP-mediated structural protection translates to functional preservation, we performed a series of physiological and behavioral assays.

**Fig. 7:**
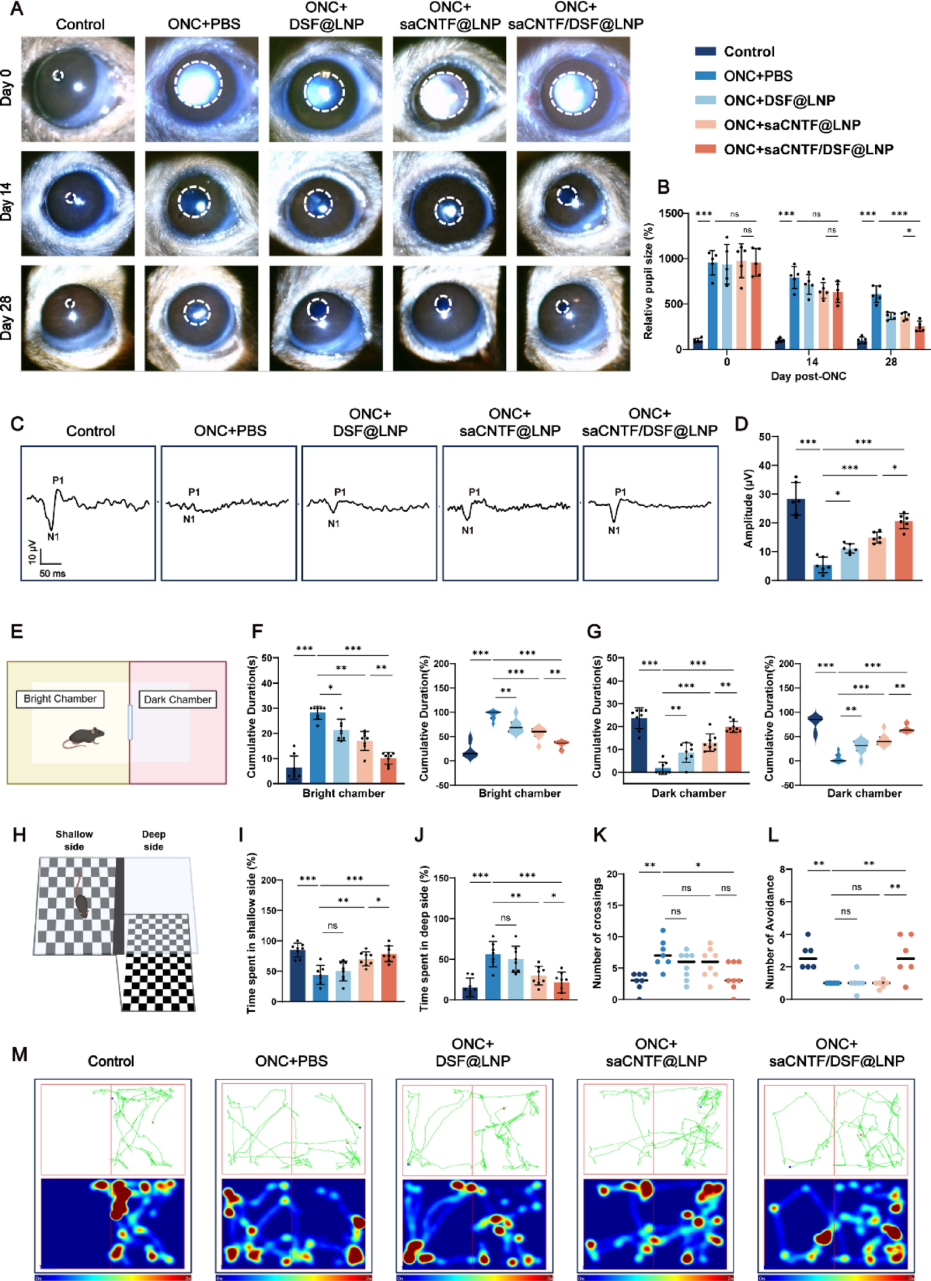
SaCNTF/DSF@LNP treatment promotes functional visual recovery after ONC. (**A**) Schematic of the pupillary light reflex (PLR) assay and representative pupil images before and after light stimulation in uninjured, ONC (PBS), and LNP-treated mice. (**B**) Quantification of pupil constriction (%) at different time points (*n* = 6). (**C**) Representative f-VEP waveforms recorded 28 days post-ONC. (**D**) Quantification of f-VEP N1-P1 amplitudes (*n* = 6). (**E**) Schematic of the light-dark box (LDB) test performed at 28 days post-ONC. (**F**, **G**) Time spent and percentage occupancy in the bright chamber (**F**) and dark chamber (**G**). (**H**) Schematic of the visual cliff test at 28 days post-ONC. Percentage of time spent in the shallow side (**I**) and deep side (**J**). (**K**) Number of crossings between sides (*n* = 8). (**L**) Number of avoidance behaviors (*n* = 6). (**M**) Heatmaps and trajectory plots showing spatial occupancy of mice. Red indicates high occupancy; blue indicates low occupancy. Data are mean ± SD, ^*^P < 0.05, ^**^P < 0.01, and ^***^P < 0.001. ns, Not significant

Pupillary light reflex (PLR) serves as a key objective indicator for assessing the integrity of the visual pathway, reflecting the capacity of RGCs to relay light signals to the brain [44]. PLR was first assessed to evaluate autonomic visual pathway function (Fig. 7A). In uninjured controls, light stimulation induced a rapid pupil contraction of ~90%, and the resultant pupil diameter was 10% of the original size. In contrast, mice with ONC injury exhibited severely impaired PLR. As expected in this model of incomplete injury, a degree of spontaneous functional recovery was observed over time, attributable to mechanisms such as spared axons and neural plasticity. The untreated ONC group showed a contraction of only ~20% on day 14 and a partial recovery to ~40% contraction by day 28, indicating substantial visual dysfunction (Fig. 7B). Given this inherent baseline recovery, our primary efficacy analysis focused on between-group comparisons at matched time points to isolate the additive therapeutic benefit. Compared to the ONC-only group, saCNTF/DSF@LNP treatment resulted in significantly greater functional restoration, achieving 37% contraction by day 14 and further improving to 75% by day 28 (Fig. 7B). This outcome was superior to that observed with DSF@LNP (63%) or saCNTF@LNP (64%) alone. Furthermore, supplementary within-group analyses (comparing post-treatment to pre-injury baselines) confirmed that the magnitude of recovery in the saCNTF/DSF@LNP group significantly exceeded the natural reparative course observed in untreated controls (Fig. S26). Overall, these results demonstrate that saCNTF/DSF@LNP treatment effectively promotes functional recovery of the visual pathway following injury.

We next evaluated the functional integrity of the retinocortical pathway using flash visual evoked potential (f-VEP), a highly sensitive electrophysiological measure of signal transmission from the retina to the visual cortex. f-VEP recordings were performed at 4 weeks post-injury (Fig. 7C). The N1-P1 amplitude, a key parameter reflecting the synchronized activity of viable retinal ganglion cells and their central projections, was significantly greater in saCNTF/DSF@LNP-treated mice (20.6 ± 2.633 µV) than in the ONC group (5.404 ± 2.712 µV) and approached levels near the sham control (28.36 ± 5.671 µV), indicating substantial functional preservation of signal conduction (Fig. 7D). In summary, saCNTF/DSF@LNP-mediated f-VEP recovery indicates restored visual pathway function, driven by enhanced RGC survival and axonal regeneration.

To assess innate visual-motor behavior, we subjected mice to the light-dark box (LDB) test at 4 weeks post-ONC (Fig. 7E). This paradigm evaluates light-avoidance behavior, an instinctual response dependent on basic light perception [45]. Mice with severe visual impairment from ONC injury spent 94.2% of the time in the bright chamber—significantly higher than the 21.2% in sham controls (*p* < 0.001). This behavioral aberration is likely attributable to their inability to reliably locate and traverse the connecting doorway to the dark chamber due to visual deficits, rather than a lack of photophobia. In contrast, saCNTF/DSF@LNP treatment restored dark chamber preference to ∼66.2%, a level comparable to the 78.8% seen in controls (*p* = 0.76; Fig. 7F and G). These results indicate that saCNTF/DSF@LNP effectively restores innate light-driven avoidance behavior, reflecting a recovery of basic visual function.

Depth perception, a higher-order visual function requiring cortical integration, was further evaluated using the visual cliff test (Fig. 7H) [42]. Uninjured mice spent only 15.3% of time on the deep side, whereas 4 weeks post-ONC-injured mice remained there 56.1% of the time, indicating a severe deficit in depth discrimination (*p* < 0.001). saCNTF/DSF@LNP treatment significantly reduced deep-side residence to 21.6% (*p* = 0.90 vs. control; Fig. 7I and J), decreased shuttle frequency (Fig. 7K), and enhanced avoidance behavior toward the cliff edge (Fig. 7L), as visualized by trajectory plots and heatmaps (Fig. 7M). This behavioral recovery demonstrates that saCNTF/DSF@LNP treatment not only restores basic light sensation but also facilitates the functional reinstatement of complex depth perception.

Taken together, these results indicate that saCNTF/DSF@LNP treatment promotes a comprehensive recovery of visual function, from basic pupillary reflexes and cortical electrophysiological responses to complex light-avoidance behavior and depth perception, demonstrating that saCNTF/DSF@LNP-mediated RGC protection and axonal regeneration contribute to the preservation of functional vision.

### Biosafety evaluation

Examination of H&E-stained sections revealed no significant tissue lesions or abnormalities in the heart, liver, spleen, lung, kidney, or hippocampus in any intervention group compared to the control group (Fig. S27). Similarly, retinal thickness showed no differences among the ONC, all treatment groups, and the normal control (Fig. S28). In the blood routine examination of mice in the normal group and saCNTF/DSF@LNP treatment group, there were no abnormalities in red blood cell count (RBC), white blood cell count (WBC), hemoglobin content (HGB), and platelet count (PLT) (Fig. S29). These results collectively affirmed the superior biocompatibility of nanoparticles in vivo.

## Discussion

TON is challenged by the absence of safe and effective therapies, often resulting in poor functional recovery [46]. The regeneration of RGCs is hindered by both their weak intrinsic regenerative ability and a profoundly inhibitory inflammatory microenvironment [25]. Restoring neuro-immune balance has therefore emerged as a critical strategy for functional repair [47]. Our study found a correlation between GSDMD-mediated microglial pyroptosis and intraocular inflammation in a TON mouse model, which were identified as key drivers of secondary inflammation and RGC degeneration, perpetuating a vicious cycle that impedes functional recovery. To address this, we developed a co-delivery LNP platform encapsulating DSF and saRNA encoding CNTF, to synergistically mitigate microglial pyroptosis while promoting RGC survival and axonal regeneration. The co-delivery system leveraged the phagocytic activity of retinal microglia for its targeted delivery [48, 49]. Moreover, this dual-target strategy showed significant inhibition on NLRP3/CASP1/GSDMD-dependent pyroptotic cell death, thereby suppressing the release of pro-inflammatory cytokines and ameliorating the inflammatory microenvironment, while CNTF expression activates downstream neurotrophic signaling pathways essential for RGC protection and axonal regrowth.

In previous studies, microglia overactivation has been considered as the main post-TON phenotype, but the ultimate fate of the microglia and its correlation with the pro-inflammatory microenvironment remains unclear [3, 50]. In this work, we identified microglial pyroptosis as a key mechanistic driver of neuroinflammation following TON. Mechanistically, we demonstrated that traumatized injury activates pyroptosis through both PIEZO1-mediated calcium influx and canonical NLRP3 inflammasome pathways, triggering Caspase1-GSDMD-mediated pyroptosis and release of pro-inflammatory cytokine IL-1β. Our engineered LNP system counteracts this cascade by delivering DSF, which directly inhibits GSDMD pore formation, thereby suppressing pyroptosis initiation and inflammatory amplification [22]. It should be noted, however, that the direct transfer of pyroptotic vesicles from microglia to RGCs and their functional consequences remain to be fully elucidated and represent an important direction for future research.

Prior studies have explored the delivery of CNTF using recombinant proteins [51] or adeno-associated virus (AAV)-based gene therapy [52]. However, these approaches face significant limitations: recombinant CNTF protein has a short half-life and lacks tissue specificity [26], while AAV vectors pose risks such as immunogenicity, limited packaging capacity, and potential genomic integration leading to mutagenesis and unpredictable long-term expression [53]. In contrast, we employed a microglia-targeted LNP system delivering saRNA encoding CNTF. Unlike conventional mRNA, saRNA leverages a viral-derived replication machinery to enable prolonged and abundant gene expression from a significantly lower dose, substantially improving both the durability and safety profile of therapeutic protein production [27, 28]. This platform enables sustained endogenous CNTF expression within the inflammatory niche and leverages the phagocytic activity of microglia for localized delivery. By targeting microglia as upstream regulatory hubs in the pathological cascade, this strategy creates a critical therapeutic time window conducive to RGC survival and axonal regeneration.

The limitations of previous therapeutic strategies using either CNTF delivery or anti-inflammatory agents underscore the critical need for a combinatorial strategy [46, 54, 55]. Our study demonstrates that coordinated intervention, simultaneously inhibiting microglial pyroptosis via DSF and providing neurotrophic support via saRNA-encoded CNTF, is essential to disrupt neuroimmune imbalance and enable significant functional recovery. The use of saRNA extends CNTF expression duration and reduces dosing frequency, overcoming key limitations of protein and virus-based gene therapy. Together, the potent inhibition of the pyroptotic cascade and sustained activation of pro-regenerative pathways position this system as a versatile and clinically promising platform for neuroinflammatory and neurodegenerative conditions beyond TON.

Despite the promising outcomes, several limitations of this study should be acknowledged. While the LNP platform demonstrates favorable biocompatibility and delivery efficiency, comprehensive assessments of its long-term safety, immune compatibility, and potential off-target effects are warranted for intravitreal administration [56]. Future efforts will focus on exploring the non-invasive delivering approach by ultrasound and optimizing the specificity and safety of the delivery system through surface-modified ligands, validating intercellular signaling mechanisms, and advancing toward clinical translation.

## Conclusions

In conclusion, we have developed a targeted nanotherapeutic platform that synergistically inhibits microglial pyroptosis and enhances neurotrophic support, effectively breaking the cycle of inflammation and regenerative failure in TON. This work not only offers a promising strategy for visual system repair but also paves the way to the broad potential of mRNA/small molecule co-delivery platforms for treating neuroinflammatory and neurodegenerative diseases.

### Methods

#### Reagents

Disulfiram (DSF) (Lot: 472316) was purchased from MedChemExpress (Monmouth Junction, USA;). The lipids SM102, DSPC, cholesterol, and DMG-PEG were sourced from AVT (Shanghai) Pharmaceutical Tech Co., LTD (Shanghai, China). BspQ1 restriction enzyme was acquired from New England Biolabs (Ipswich, USA). The DNA extraction kit was from Vazyme (Nanjing, China). mRNA synthesis kit and magnetic purification beads were obtained from SynthGene (Nanjing, China). Amicon^®^ Ultra Centrifugal Filters were purchased from Merck Millipore (Burlington, USA). Cell Counting Kit-8 (CCK-8) (Lot: WK710) and primary antibodies against Iba1, βIII-tubulin, and GAP43 were purchased from Abcam (Cambridge, UK). The LDH Assay Kit was from Promega (Madison, USA). RiboGreen dye, Ethidium Homodimer-1 (EthD-1) (Lot: 2326049), Alexa Fluor 647-conjugated phalloidin (Lot: 2575967), DAPI (Lot: ZC4229401), and live/dead dye were procured from Invitrogen (Carlsbad, USA). The Papain Dissociation System was from Worthington (Lakewood, USA). Fluo-4 AM and Dihydroethidium (DHE) were obtained from Beyotime (Shanghai, China). D-luciferin potassium was from APExBIO (Houston, USA). Alexa Fluor 555-Conjugated Cholera Toxin Subunit B (CTB), ECL substrate, and the BCA assay kit were purchased from Thermo Fisher Scientific (Waltham, USA). The Mouse IL-1β ELISA Kit was from R&D Systems (Minneapolis, USA). Primary antibody against RBPMS (Lot: 00172689) was from ProteinTech (Wuhan, China); beta-Actin (Lot: 19), Gasdermin D (Lot: 4), DYKDDDDK Tag (Lot: 7), IL-1 beta (Lot: 1), was from Cell Signaling Technology (Danvers, USA). Primary antibodies for immunoblotting and Alexa Fluor 488-conjugated secondary antibody were from Cell Signaling Technology (Danvers, USA). The protease inhibitor cocktail was from New Cell & Molecular Biotech (Suzhou, China). DMEM (Lot: 6125504), RPMI-1640 medium (Lot: 6125306), Fetal Bovine Serum (FBS) (Lot: B210740RP), Penicillin-Streptomycin (Lot: 242480), and 0.25% Trypsin-EDTA (Lot: 2919428) were purchased from Gibco (Thermo Fisher Scientific, Waltham, USA).

#### Bulk RNA-Seq

RNA was extracted from each adult mouse retina sample using Trizol, and quality control was performed using the Bioanalyzer 2100 system (Agilent Technologies, Santa Clara, USA). RNA sequencing (RNA-Seq) libraries were then prepared and sequenced using the Illumina platform. The raw data, in FASTQ format, were initially processed with the ‘fastp’ software to remove reads containing adapters, poly-N sequences, and low-quality data, resulting in clean reads. Following this, ‘HISAT2’ (v2.0.5) was used to align the clean reads to the reference genome, and ‘featureCounts’ package (v1.5.0) was employed to count the number of reads mapped to each gene. The ‘decoupler’ package (version 2.1.1) was employed to identify differentially expressed genes (DEGs) between the control and ONC groups with the criterion of adjusted p value < 0.05 and |log2FC|> 1 using the ‘DESeq2’ method. In addition, the enrichment scores of Gene Ontology (GO) terms across the blank, nigericin, and nigericin with saCNTF/DSF@LNP treatment groups were calculated based on this package. For gene enrichment analysis, the R package ‘ClusterProfiler’ (version 4.16.0) was utilized to identify pathways that are enriched in the ONC group, referencing the gene set from the Molecular Signatures Database (MSigDB). The biological terms or pathways with adjusted p-value < 0.25 was considered statistically significant enrichment and visualized by the ‘GseaVis’ R package (version 0.1.1).

#### scRNA-Seq

A single-cell transcriptomics dataset of adult mouse retina before and at six time points after ONC injury was downloaded from the Gene Expression Omnibus (GEO) database (accession numbers: GSE199317). Subsequently, the gene expression values preprocessed by the original article were used to perform downstream analysis. The expression levels of NLRP3, Caspase1, GSDMD, IL-1β and Piezo1 across various time points and cell subpopulations before and at six time points after ONC injury were visualized by bubble plots and violin plots for representation with the ‘OmicVerse’ framework (version 1.7.5). The ‘CellChat’ R package (version 2.1.2) and “CellPhoneDB” package (version 2.1.2) were employed to analyze cell-cell interactions, elucidating the communication relationships and their strengths among different cell types in the retina.

#### mRNA Synthesis

The Flag-tagged mouse CNTF-encoding gene (Gene ID: 12803) was inserted into commercial linear mRNA and saRNA backbone vector (VectorBuilder, Guangzhou, China) for in vitro transcription (IVT), respectively. Both plasmids were linearized by BspQ1 (New England Biolabs, USA) and purified using a DNA extraction kit according to the manufacturer’s instructions. The linearized DNA templates were then processed for mRNA IVT using a one-step T7 co-transcription RNA synthesis kit by mixing with transcription buffer, ATP, GTP, CTP, m1ψ, Cap GAG, and T7 RNA polymerase. Cap GAU was used alternatively for saRNA synthesis. After incubation at 37 °C for 2 h, DNase I was added to remove the DNA template and incubated for an additional 20 min. The mRNA and saRNA products were further purified by poly-T-conjugated magnetic RNA purification beads. The removal of the DNA template was verified by qPCR. The quality control of CNTF mRNA and saRNA was tested using NanoDrop (Thermo Fisher Scientific, Waltham, USA) and agarose gel electrophoresis.

#### LNP Preparation

Ionize lipid SM102, DSPC, cholesterol, and DMG-PEG were dissolved in ethanol at 10 mM and mixed at the molar ratio of 50:10:38.5:1.5. For DSF-incorporated RNA LNPs, DSF was diluted by the above lipid mixture so that the mass ratio of DSF: lipids reached 1:4. The ethanol phase containing the lipid mixture with or without DSF was added to the water phase containing CNTF mRNA and CNTF saRNA diluted in 25 mM NaAc (pH 4.0), respectively, under vigorous vortex for 30 s. The mass ratio of RNA to lipids was 1:20. The RNA-encapsulated LNPs were then purified using Amicon^®^ Ultra Centrifugal Filters (MWCO 100 kDa) to remove ethanol and to exchange the LNP storage buffer into the Tris-Sucrose (pH 7.4) cryo-protective buffer. The DSF LNPs were prepared using the same method without RNA encapsulation. The particle size and the polydispersity index (PDI) of all the LNPs were measured by DLS (Malvern Panalytical, Malvern, UK). The RNA encapsulation efficiency was measured using RiboGreen. The LNPs were aliquoted at a final RNA concentration of 0.5 µg/µL and preserved at −80 °C till further use.

#### Cell Culture

R28 (RRID: CVCL_5I35) and BV2 (RRID: CVCL_0182) cells were purchased from Anwei-sci Cell Center (Shanghai, China) and Fuheng Biotechnology Co., Ltd (Shanghai, China), respectively. All experiments were conducted using these cells, which were certified free of contamination and authenticated by Short Tandem Repeat (STR) profiling. Cells were cultured in 37 °C incubator with 5% CO_2_ using DMEM and RPMI-1640 medium, respectively, containing 10% FBS and 1% Penicillin-Streptomycin antibiotics. Cells were passaged using 0.25% Trypsin-EDTA when reaching 90% attachment. All in vitro experiments were performed with at least three independent biological replicates to ensure reproducibility and statistical power.

#### In Vitro CNTF expression

BV2 cells were seeded into 12-well plates at a density of 2.5 × 10^5^ cells and cultured at 37 °C with 5% CO₂ overnight before being transfected with CNTF@LNP, CNTF/DSF@LNP, saCNTF@LNP, and saCNTF/DSF@LNP, respectively, for 48 h. For the comparison of CNTF and saCNTF expression, R28 cells were transfected with CNTF@LNP and saCNTF@LNP, respectively, for 24, 48, and 72 h. Cell lysates were collected for immunoblotting analysis to detect Flag-tagged CNTF expression level.

#### Cell viability assay

Cell viability was evaluated using the Cell Counting Kit-8. BV2 Cells were seeded into 96-well plates at a density of 8 × 10^3^ cells per well and cultured at 37 °C with 5% CO₂ overnight. Cells were then treated with equivalent concentrations of DSF or DSF@LNP for 24 h before being incubated with CCK-8 solution at 37 °C for 1 h. Absorbance at 450 nm (A_450_) was measured using a microplate reader (Biotek, Winooski, USA). The viability of treated cells was calculated as follows: Viability (%) =A_450_ (treatment)/A_450_ (control) × 100%. All experiments were performed with at least three independent replicates per treatment. Statistical analysis was carried out using GraphPad Prism 10.

#### Pyroptosis analysis

To induce pyroptosis and treatment, BV2 cells were pretreated with 10 µg/mL DSF, DSF@LNP, or saCNTF/DSF@LNP for 12 h, followed by stimulation with 20 µM nigericin for 6 h. Pyroptosis was assessed by measuring lactate dehydrogenase (LDH) release and Ethidium Homodimer-1 (EthD-1) staining. Culture supernatants from cells with indicated treatments were collected for LDH measurements using the LDH Assay Kit according to the manufacturer’s instructions at A_450_. Cells were stained with 2 µM EthD-1 for 20 min at 37 °C, followed by PBS washing. Fluorescence images were acquired using a fluorescence microscope (Nikon, Minato City, Japan).

#### Immunoblotting

Cells or tissues were lysed in RIPA buffer with protease inhibitor cocktail. The lysates were centrifuged to remove debris, and supernatants were collected for protein quantification using a BCA assay kit. Samples are then denatured with SDS-based loading buffer at 100 °C for 10 min. Electrophoresis was conducted, followed by electro-transfer onto a PVDF membrane, which was blocked with skimmed milk for 2 h, incubated with primary antibodies overnight, and exposed to HRP-conjugated secondary antibodies. The chemiluminescence signals were detected using ECL Substrate and Odyssey^®^ XF Imaging System. Image analysis was performed using ImageJ^®^.

#### Cytokine measurement

A transwell co-culture system was established with BV2 cells in the upper chamber and R28 cells in the lower chamber. Supernatants from cells under the indicated treatments in both chambers were collected, and concentrations of IL-1β were measured by Mouse IL-1β ELISA Kit according to the manufacturer’s instructions. A_450_ was measured using a microplate reader (Biotek, Winooski, USA). Concentrations were calculated based on a standard curve and expressed as mean ± SD from three independent experiments.

#### Immunofluorescence

Cells cultured on glass coverslips were fixed with 4% paraformaldehyde for 15 min at room temperature. After washing with PBS, the cells were permeabilized with 0.1% Triton X-100 for 10 min and blocked with 5% BSA for 1 h. Primary antibodies were applied overnight at 4 °C, followed by PBS wash and incubation with the corresponding secondary antibodies at room temperature for 1 h. After being rewashed by PBS, cells were incubated with Alexa Fluor 647-conjugated phalloidins (Invitrogen, Carlsbad, USA) and DAPI (Invitrogen, Carlsbad, USA) for cytoskeleton and nucleus staining, respectively. Fluorescence images were immediately captured using an LSCM (Leica, Germany).

#### Animals

C57BL/6 mice (8 weeks old) were housed under a 12 h light/dark cycle with free access to food and water. All animal procedures were approved by the Animal Ethics Committee of the Ninth People’s Hospital, Shanghai Jiao Tong University School of Medicine (Approval No. SH9H-2025-A27-1).

#### ONC model and intravitreal injection

C57BL/6 mice (8 weeks old) were anesthetized vima intraperitoneal injection of Zoletil and dexmedetomidine. The optic nerve crush (ONC) was performed unilaterally in the left eye. A lateral canthotomy was performed to expose the intraorbital optic nerve. The nerve was then crushed for 10 s using cross-action forceps, which ensures a uniform and consistent force across all animals. The crush site was located approximately 1 mm from the optic disc. The efficiency and severity of the ONC injury are assessed through immediate indicator, primary functional metric and ancillary confirmation. For intravitreal injections, a glass micropipette was inserted through the pars plana to deliver 2 µL of therapeutics into the vitreous body. Ophthalmic ointment was applied to protect the cornea after surgery.

#### Calcium influx assay

Retinas harvested from both control mice and ONC-injured mice at 4 days post-injury were dissociated into single-cell suspensions by using the Papain Dissociation System (Worthington, Lakewood, USA). After passing through a cell strainer to remove undissociated tissue aggregates, cells were incubated with 1 µM Fluo-4 AM for 30 min at 37 °C, and fluorescence was analyzed by flow cytometry (Beckman Coulter, Brea, USA).

#### In Vivo expression and microglia-targeting assay

Mice were injected intraperitoneally with 1 µg LNP-encapsulated Luciferase mRNA or Luciferase saRNA reporter, respectively. Then, D-luciferin potassium was injected intraperitoneally at 150 mg/kg. Bioluminescence was analyzed using an in vivo imaging system (IVIS, PerkinElmer, Waltham, USA).

For in vivo microglia-targeting assay, ONC-injured mice received an intravitreal injection of 1 µg EGFP mRNA-loaded LNPs. Retinas were harvested at 24 h post-injection to prepare single-cell suspensions, which were then stained with anti-CD11b-PE-eFluor 610 and FVD eFluor 506 Live/Dead dye. EGFP fluorescence intensity in CD11b⁺ and CD11b⁻ cells was analyzed by flow cytometry (Beckman Coulter, Brea, USA) and FlowJo^®^ software.

#### Tissue fluorescence staining

For the detection of retinal ROS level, freshly enucleated eyeballs from each group were embedded in OCT compound and rapidly frozen. Unfixed cryosections (10 μm thickness) were incubated with 10 µM dihydroethidium (DHE) solution at 37 °C for 30 min. After washing with PBS, fluorescence images were acquired under identical microscope settings for both groups. ROS fluorescence intensity was quantified and compared between ONC and control retinas using ImageJ software.

For retina immunofluorescence staining, animals were perfused transcardially sequentially with ice-cold PBS and 4% PFA. After optic nerve transection near the chiasm, eyeballs were dissected and post-fixed in 4% PFA. Retinal whole mounts were blocked in PBS containing 5% normal donkey serum and 0.1% Triton X-100 for 1 h, followed by overnight incubation with primary antibodies: anti-RBPMS or anti-Iba1, respectively, at 4 °C. After washing with PBS, samples were incubated with Alexa Fluor 488-conjugated secondary antibody for 2 h at room temperature, rewashed, and mounted with Fluoromount-G. Imaging was performed on a laser scanning confocal microscope (LSCM, Zeiss, Jena, Germany). Central and peripheral retinal regions were sampled for RGC quantification and analysis.

For the detection of neuroaxonal growth, 2 µL of Alexa Fluor 555-CTB was injected intravitreally. After 72 h, mice were perfused with 4% PFA. Optic nerves were dissected, sectioned, and imaged using a microscope (NIKON, Minato City, Japan).

For neuroaxonal immunofluorescence staining, surgical removal of optic nerves from perfused mice and posterior fixation with 4% PFA. Stained with anti-βIII-tubulin protein and GAP43, and imaged under a microscope (NIKON, Minato City, Japan).

#### Histological analyses

For H&E staining, mouse eyeballs were fixed in Davidson’s fixative at 4 °C overnight, then dehydrated through a graded ethanol series, cleared, and embedded in paraffin. Sections of 5 μm thickness were cut using a microtome (RM2016, Leica Microsystems, Wetzlar, Germany), floated in ultrapure water at room temperature, flattened at 55 °C, and mounted on adhesive slides. After drying for 6 h, sections containing the optic nerve head were selected and stained automatically with hematoxylin and eosin. The slides were coverslipped with neutral balsam and imaged using a microscope (NIKON, Minato City, Japan).

For Nissl staining, tissue sections containing the optic nerve head were selected and subjected to Nissl staining using 0.1% cresyl violet solution. After differentiation and dehydration through graded alcohols, slides were cleared in xylene and coverslipped with neutral balsam. Images were captured using the same digital microscope (NIKON, Minato City, Japan).

For histological analysis of the retina, the retinal thickness of the ganglion cell layer (GCL) and the number of nuclei per column in the GCL were determined in this study. A total of positions, with each point at a 300 μm interval from the optic nerve head (ONH) and retinal margin, were selected for measurement for each section. Both the measurement at each point and the average measurement of all points were evaluated and analyzed.

#### Optical coherence tomography (OCT)

Mice were anesthetized, and pupils were dilated by tropicamide eye drops. OCT imaging (Heidelberg SPECTRALIS, Heidelberg, Germany) was performed at baseline and 4 weeks post-ONC. Circular scans centered on the optic nerve head were acquired with a diameter of 1800 μm. Cross-sectional images through the optic nerve were also obtained for reference. Ganglion cell complex (GCC) thickness was quantified using built-in software. Retinal nerve fiber layer (RNFL) and total retinal thickness were manually measured at 600 μm (center) and 1200 μm (periphery) from the optic nerve.

#### Pupillary light reflex (PLR) test

Mice were dark-adapted for 2 h prior to testing. Under light anesthesia, each mouse was restrained in a custom holder. A natural light source of consistent intensity was applied to one eye for 1 min; the stimulator fully enclosed the eye to avoid contralateral exposure. Pupil images were captured using a slit-lamp lens and analyzed with ImageJ to quantify pupil area and constriction response across groups.

#### Flash visual evoked potential (f-VEP)

f-VEP recordings were performed 4 weeks post-intervention using an Espion E3 system (Diagnosys, Lowell, USA). After overnight dark adaptation, mice were anesthetized with isoflurane and maintained at 37 °C. Pupils were dilated with compound tropicamide drops. Subdermal needle electrodes were positioned as follows: the recording electrode was placed along the midline of the scalp between the ears, aligned over the primary visual cortex; the reference electrode was inserted in the contralateral cheek; and the ground electrode was placed in the tail. White flash stimuli (1.3 Hz, 300 ms) were delivered under dim red light; the contralateral eye was covered. The N1-P1 amplitude was automatically computed from averaged waveforms.

#### Light-dark box test

Visual avoidance behavior was assessed 4 weeks post-ONC and treatment using a light-dark box under natural light. The apparatus consisted of interconnected light and dark chambers. Mouse movement was recorded with an overhead CCD camera supplemented by infrared backlighting. Time spent in each chamber and movement trajectories were analyzed using VisuTrack^®^ software.

#### Visual cliff test

Depth perception was evaluated in a clear plexiglass box (62 × 62 × 19 cm) divided into shallow and deep zones with patterned surfaces at different depths. 4 weeks post-ONC and treatment mice were placed onto the platform, and the time taken to select the deep and shallow areas was recorded. Movement paths and heatmaps were analyzed using VisuTrack^®^ software. The apparatus was cleaned between trials.

#### Biosafety assessment

Histopathological Examination: major organs (heart, liver, spleen, lung, kidney, and hippocampus) and retinal tissues were collected from each experimental group at the endpoint of the study. Tissues were fixed in 4% paraformaldehyde, embedded in paraffin, and sectioned at a thickness of 5 μm. Hematoxylin and eosin (H&E) staining was performed according to standard protocols. Histopathological evaluation was carried out by a pathologist blinded to the treatment groups using a light microscope. Retinal thickness was measured on H&E-stained retinal sections using ImageJ^®^ software.

Hematological Analysis: blood samples were collected from the orbital venous plexus of mice in control and saCNTF/DSF@LNP-treated groups. Routine blood tests, including red blood cell count (RBC), white blood cell count (WBC), hemoglobin concentration (HGB), and platelet count (PLT), were performed using an automated hematology analyzer.

#### Quantitative RT-PCR

Total RNA from cells or tissues was extracted using Trizol, and then reverse transcribed into cDNA. qRT-PCR analysis was performed using PrimeScript RT Reagent Kit (Perfect Real Time) and Roche LightCycler 480 II Fluorescence Quantitative PCR Instrument. Table S1 contains the primers for qRT-PCR specified.

#### Quantification and statistical analysis

Excel and GraphPad Prism 10 were used for statistical analysis. All the statistical details for each experiment were described in the figure legends.

For tests where injection and injury treatment were performed unilaterally on one eye for each mouse, the indicated ‘n’ represents individual eyes and thus only one eye per mouse was used for statistical analysis.

An unpaired t-test was used to compare two groups. One-way ANOVA with Tukey’s multiple comparisons test, with follow-up tests to compare each group with every other group, was used to compare multiple groups. A *P*-value ≤ 0.05 was considered statistically significant.

## Supplementary Information

Supplementary Material 1

## Data Availability

The bulk RNA-seq data generated in this study are available upon request from the corresponding author. The scRNA-seq data utilized herein were downloaded from the GEO database (accession number GSE199317), https://www.ncbi.nlm.nih.gov/geo/query/acc.cgi? acc=GSE199317. Additional information necessary to reanalyze the data reported in this paper is available from the corresponding author upon reasonable request.

## Abbreviations

TON: Traumatic optic neuropathy
ONC: Optic nerve crush
DSF: Disulfiram
CNTF: Ciliary neurotrophic factor
saCNTF: Self-amplifying RNA encoding CNTF
GSDMD: Gasdermin D
NLRP3: NOD-like receptor family pyrin domain containing 3
CASP1: Caspase1
PIEZO1: Piezo-type mechanosensitive ion channel component 1
Iba-1: Ionized calcium-binding adapter molecule 1
LNP: Lipid nanoparticle
RGC: Retinal ganglion cell
GCL: Ganglion cell layer
RNFL: Retinal nerve fiber layer
ROS: Reactive oxygen species
scRNA-seq: Single-cell RNA sequencing
Fluc: Firefly-luciferase
EGFP: Enhanced green fluorescence protein
