# Supplementary material for: Self-amplifying RNA therapy encoding CNTF with disulfiram co-delivery promotes optic nerve repair through microglial pyroptosis inhibition and RGC axonal regeneration
Source: J Nanobiotechnology. 2026 Mar 13;24:378. doi: 10.1186/s12951-026-04272-x (PMC13101238; doi:10.1186/s12951-026-04272-x)
Supplement: Supplementary file 1 — Supplementary Material 1 [file 12951_2026_4272_MOESM1_ESM.docx]

Supplementary Information

**Self-Amplifying RNA Therapy Encoding CNTF with Disulfiram Co-delivery Promotes Optic Nerve Repair Through Microglial Pyroptosis Inhibition and RGC Axonal Regeneration**

Qianyue Zhang^1†^, Yusha Liu^1†^, Qin Wei^1†^, Mingyang Song^1^, Siwei Liu^1^, Haiyang Zhang^1^, Tingyu Deng^1^, Chutong Zhang^1^, Kexin Tan^1^, Rui Huang^1^, Ni Ni^1^, Jun Zhang^1^, Ping Gu^1^, Gang Du^2,3^, Jipeng Li^1*^, Yingzhi Chen^1*^, Huifang Zhou^1*^, and Xianqun Fan^1*^


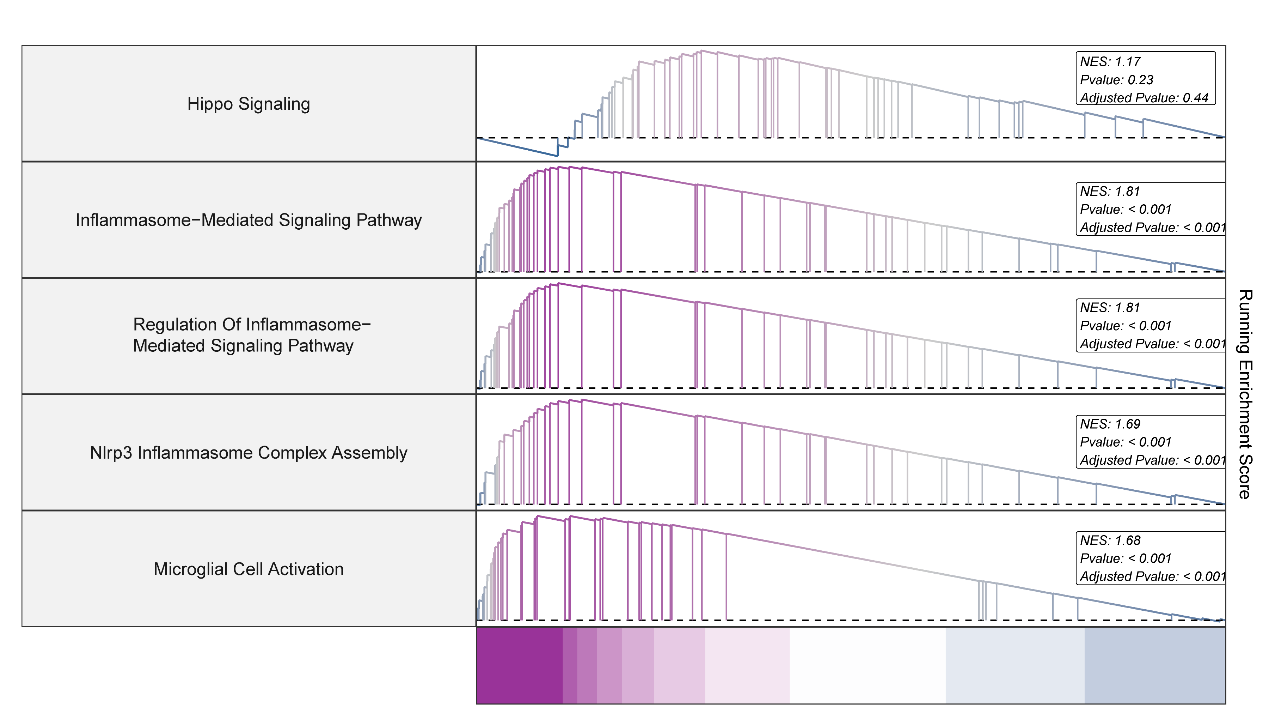


**Figure S1.** GSEA of hippo signaling pathway, inflammasome-mediated signaling pathway, regulation of inflammasome-mediated signaling pathway, Nlrp3 inflammasome complex assembly pathway, microglial cell activation pathway and pyroptotic inflammatory response pathway.


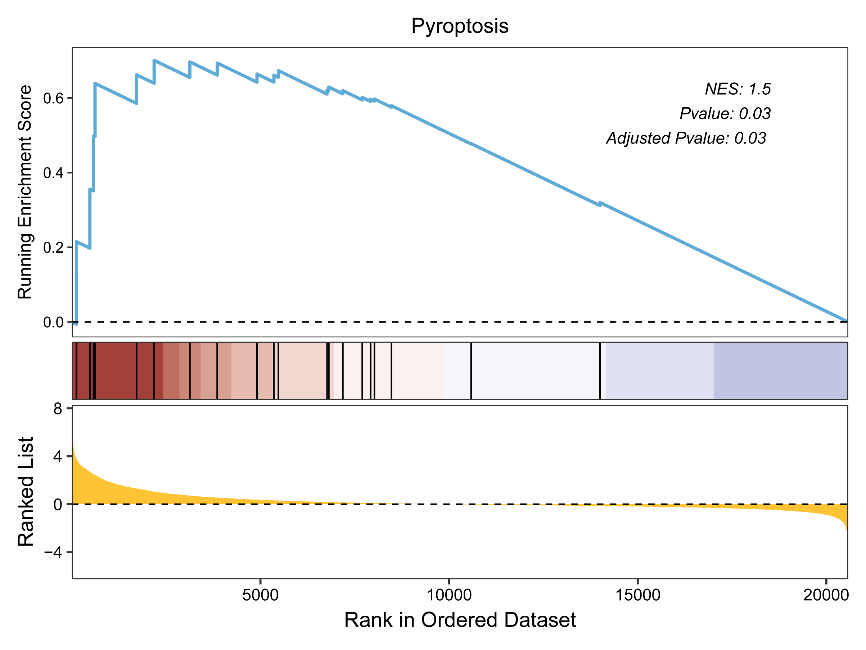


**Figure S2.** GSEA of pyroptosis pathway.


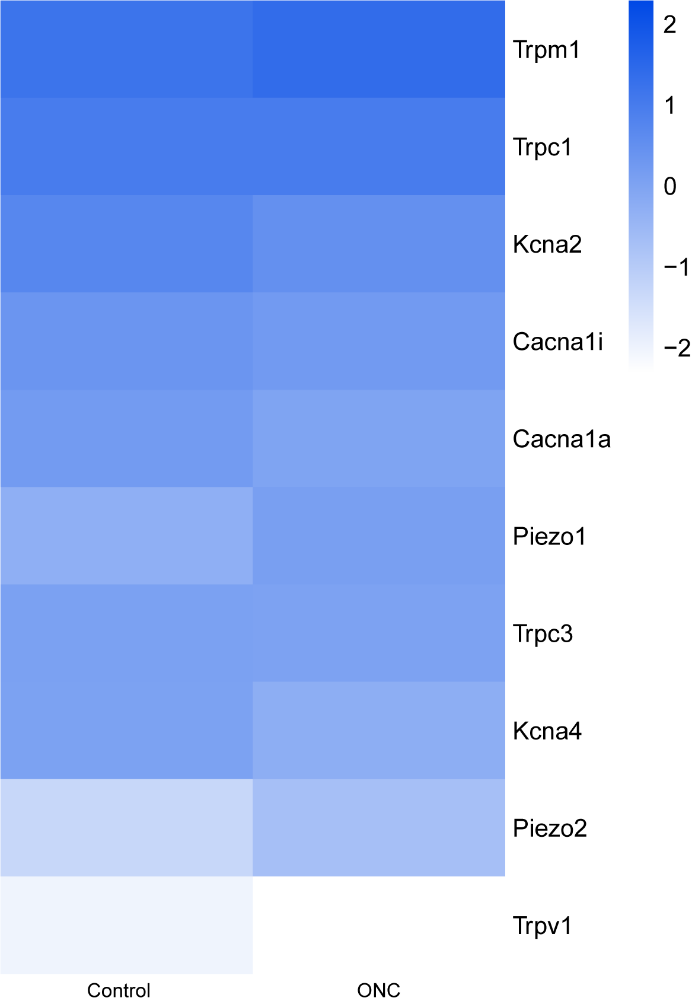


**Figure S3.** Heatmap of calcium channels expression level in retina tissues (Control versus ONC group).


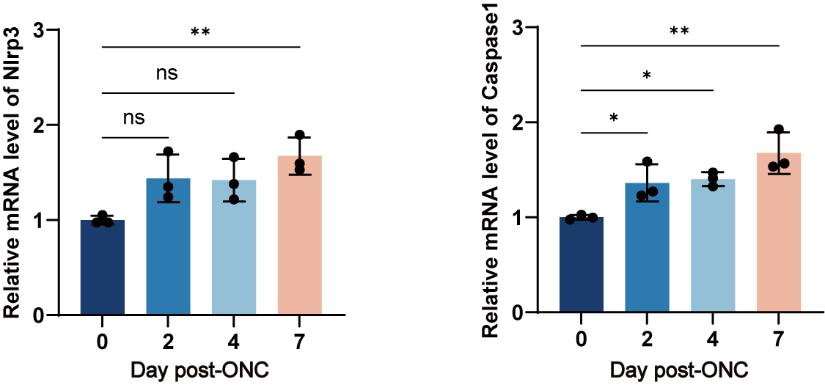


**Figure S4.** qRT-PCR analysis of NLRP3 and Caspase1 mRNA (*n* = 3).


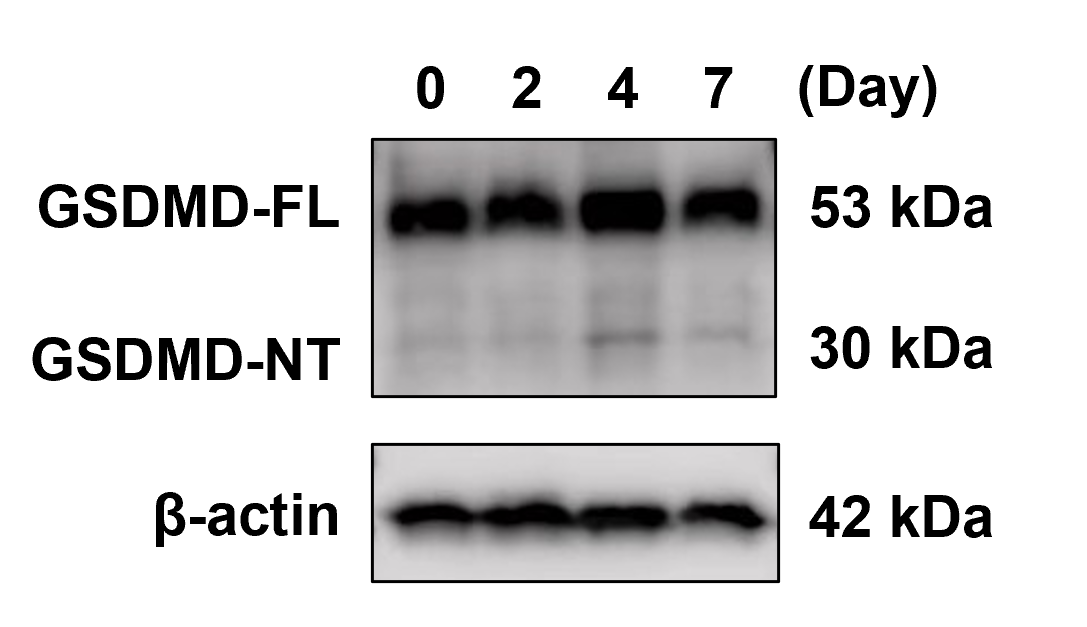


**Figure S5.** Western blot analysis showing the cleaved N-terminal fragment of GSDMD (GSDMD-NT) in retinal lysates at Days 2, 4, and 7 post-ONC compared to uninjured control. β-actin serves as a loading control.


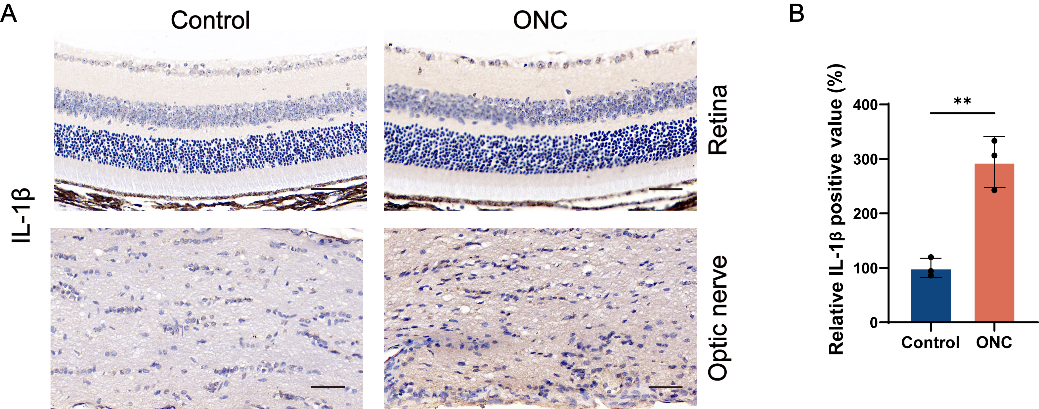


**Figure S6.**(A) Representative images of immunohistochemical staining for IL-1β at day 7 post-ONC. (B) Quantification of IL-1β-positive areas of optic nerve from mice with ONC (*n*=3). Data are presented as mean ± SD; **P < 0.01 by unpaired t-test. Scale bar = 50 μm.


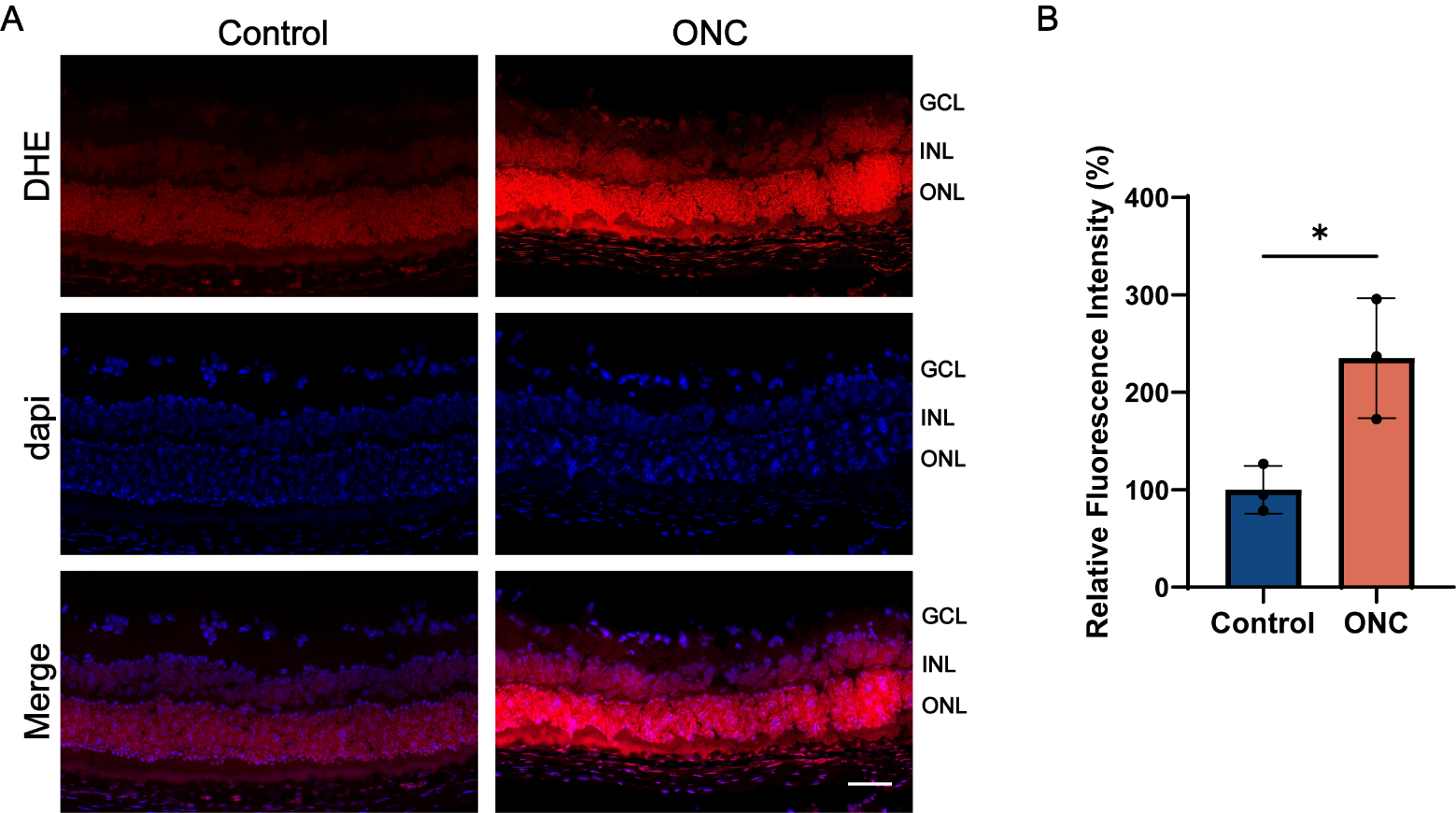


**Figure S7.** ROS increased and localized in the retina of ONC mice. (A) Representative images of dihydroethidium (DHE) staining in ONC and healthy control group. (B) Quantification of DHE-positive area (%) in retinal sections (*n*=3). Data are presented as mean ± SD; *P < 0.05 by unpaired t-test. Scale bar = 50 μm.


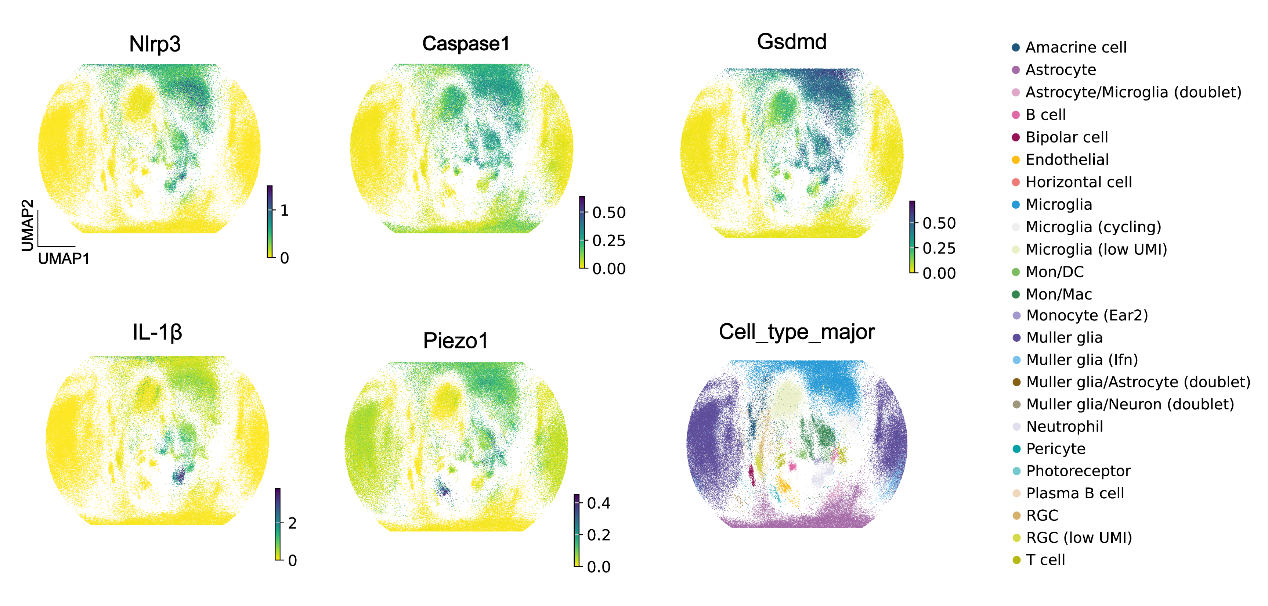


**Figure S8.** Uniform Manifold Approximation and Projection (UMAP) plots of expression levels of Nlrp3, Caspase1, Gsdmd, IL-1β, and Piezo1 in different cell types of the retina.


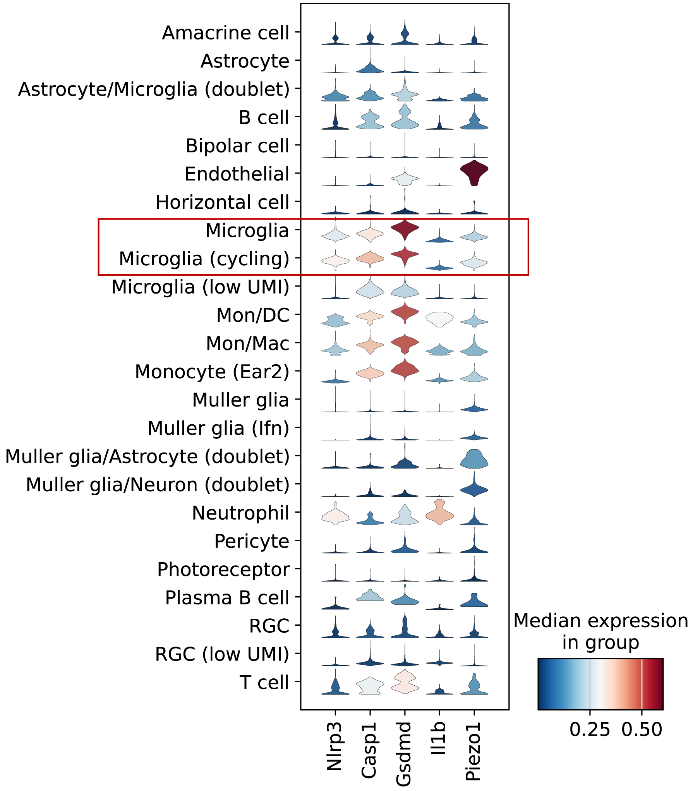


**Figure S9.** Violin plots showing the relative expression levels of Nlrp3, Caspase1, Gsdmd, IL-1β, and Piezo1 in different cell types of the retina.


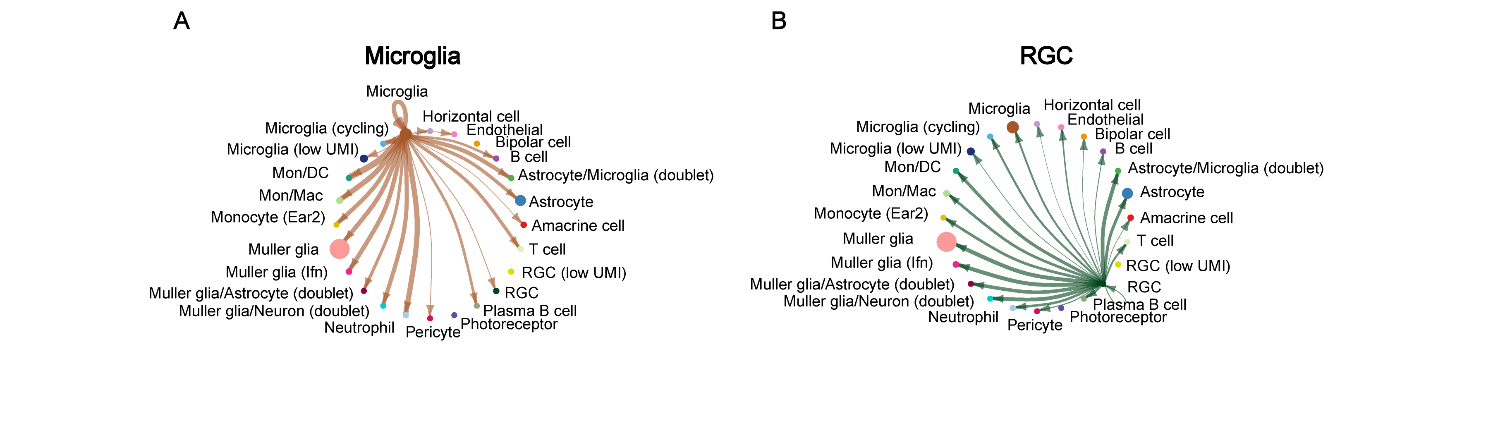


**Figure S10.** (A) The interaction net plot between the microgila cells with other cell types in retina. (B) The interaction net plot between the retinal ganglion cells (RGCs) with other cell types in retina.


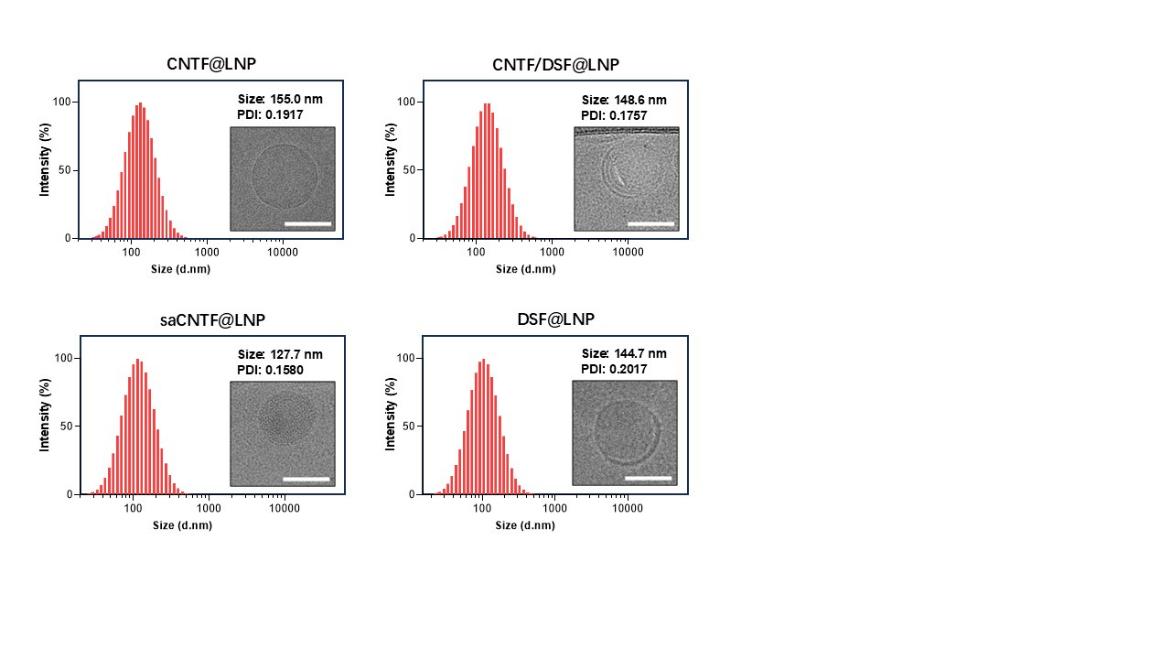


**Figure S11.** Hydrodynamic size distribution and Cryo-EM images of CNTF@LNP, CNTF/DSF@LNP, saCNTF@LNP and DSF@LNP. Scale bar = 100 nm.


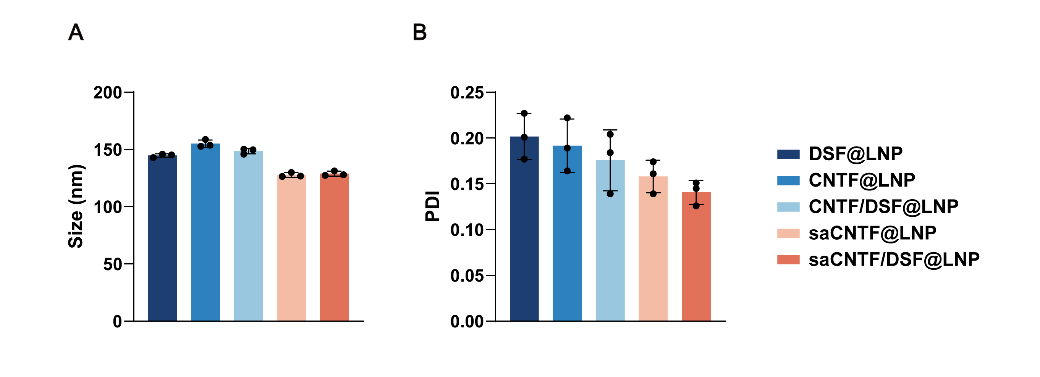


**Figure S12.** Comprehensive characterization of LNP formulations. (A) Hydrodynamic size (diameter, nm) of DSF@LNP, CNTF@LNP, CNTF/DSF@LNP, saCNTF@LNP, and saCNTF/DSF@LNP nanoparticles measured by dynamic light scattering (DLS). (B) Polydispersity index (PDI) of DSF@LNP, CNTF@LNP, CNTF/DSF@LNP, saCNTF@LNP, and saCNTF/DSF@LNP nanoparticles, as determined by DLS.


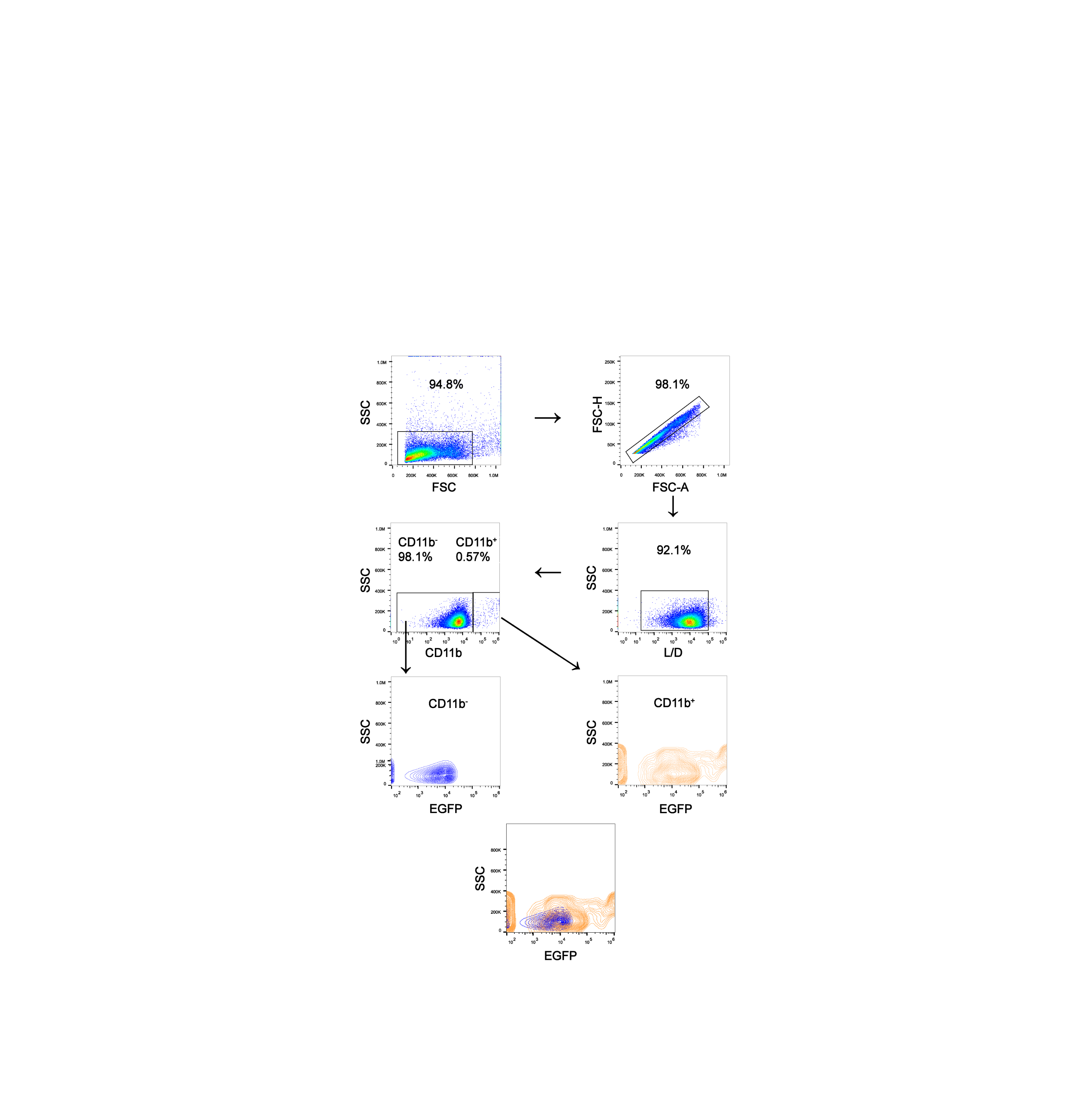


**Figure S13.** Flow cytometry analysis and gating strategy of retinal microglia targeting of EGFP mRNA-encapsulated LNPs.


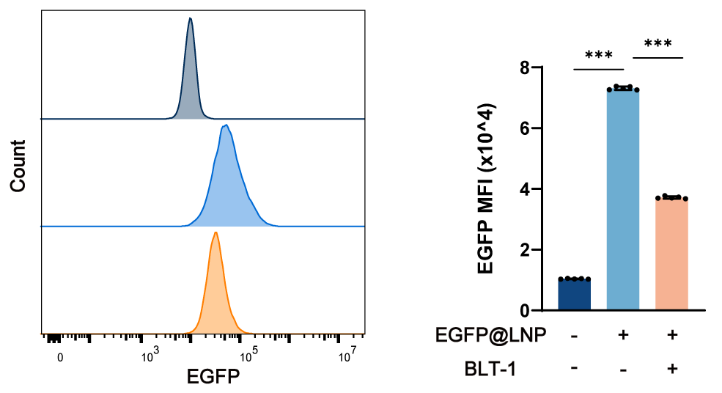


**Figure S14.** Flow cytometry analysis of EGFP expression in BV2 microglial cells from Control, EGFP@LNP and EGFP@LNP + BLT-1 (SR-B1 inhibitor, 5 µM for 1 hour) for 24 h. Representative histograms (left) and quantification of EGFP-positive cells (right) are shown (*n*=3).


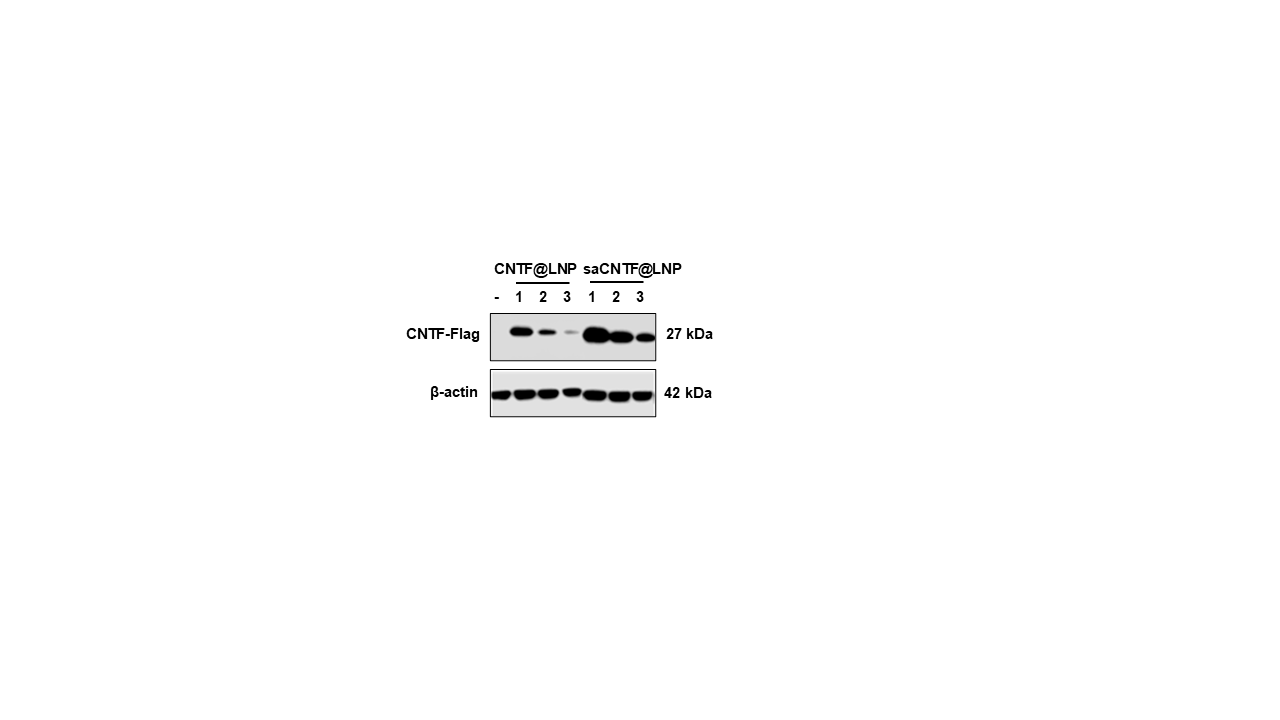


**Figure S15.** Western Blot was used to analyze the expression of CNTF protein in BV2 cells after Blank and transfection with CNTF@LNP and saCNTF/DSF@LNP for 1, 2, and 3 days.


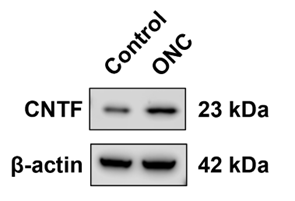


**Figure S16.** Western blot analysis of endogenous CNTF protein levels in retinal lysates at day 4 post-ONC compared to uninjured controls.


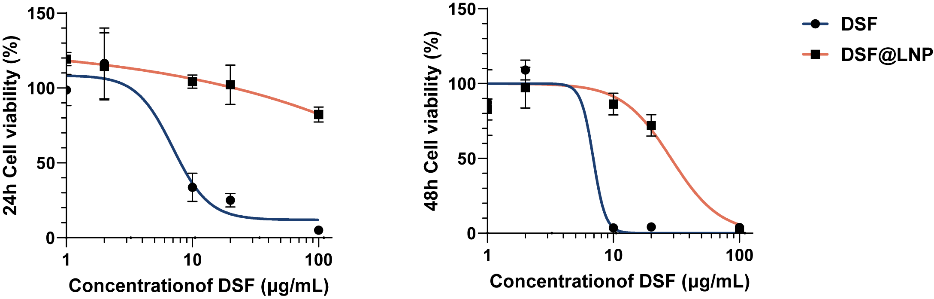


**Figure S17.** Cell viability (CCK-8 assay) of BV2 microglia treated for 48 hours with free DSF or DSF@LNP across a range of concentrations (*n*=5).


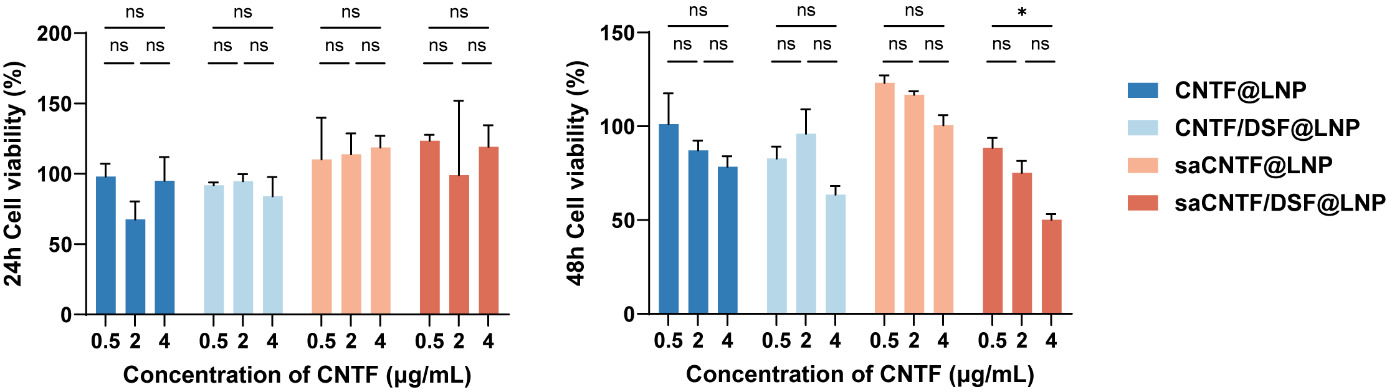


**Figure S18.** Cell viability of BV2 cells treated with CNTF@LNP, CNTF/DSF@LNP, saCNTF@LNP, or saCNTF/DSF@LNP (0.5–4 µg/mL, CNTF-equivalent) for 48 h.


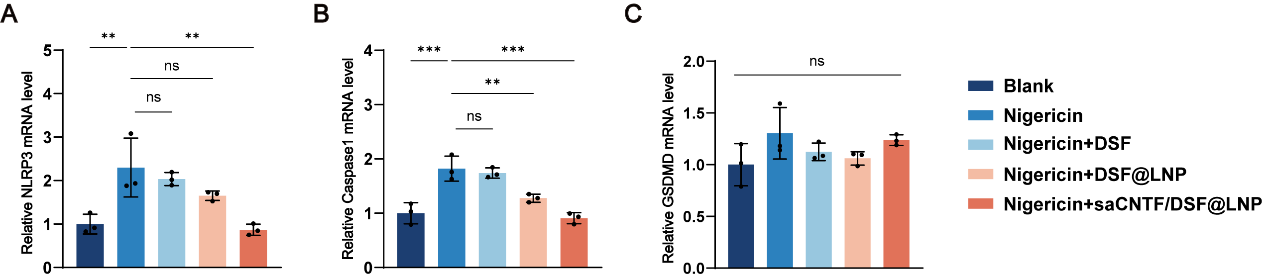


**Figure S19.** Result of pyroptosis-related indicator mRNA expression level of NLRP3, Caspase1, GSDMD (*n*=3). Data are presented as mean ± SD; one-way ANOVA with Tukey’s multiple comparisons test, **P < 0.01, ***P < 0.001, ns: the difference was not statistically significant.


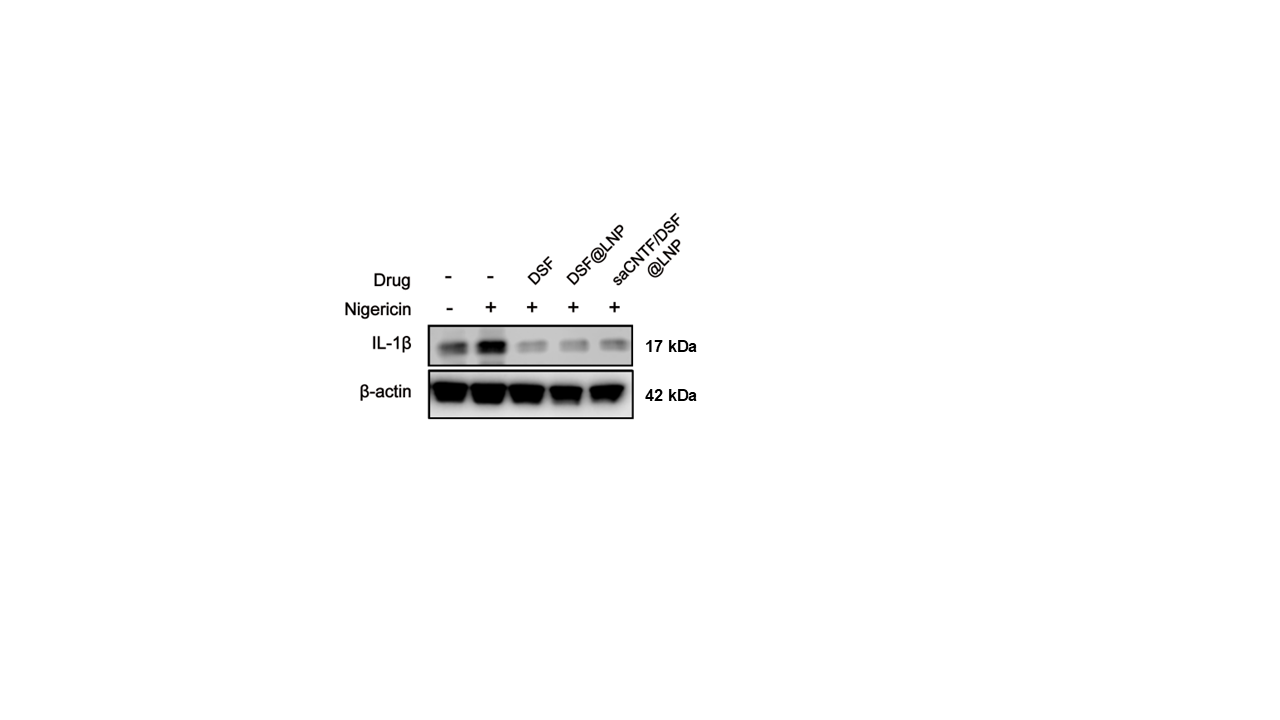


**Figure S20.** Western blot analysis of IL-1β expression in BV2 cell lysates.

**
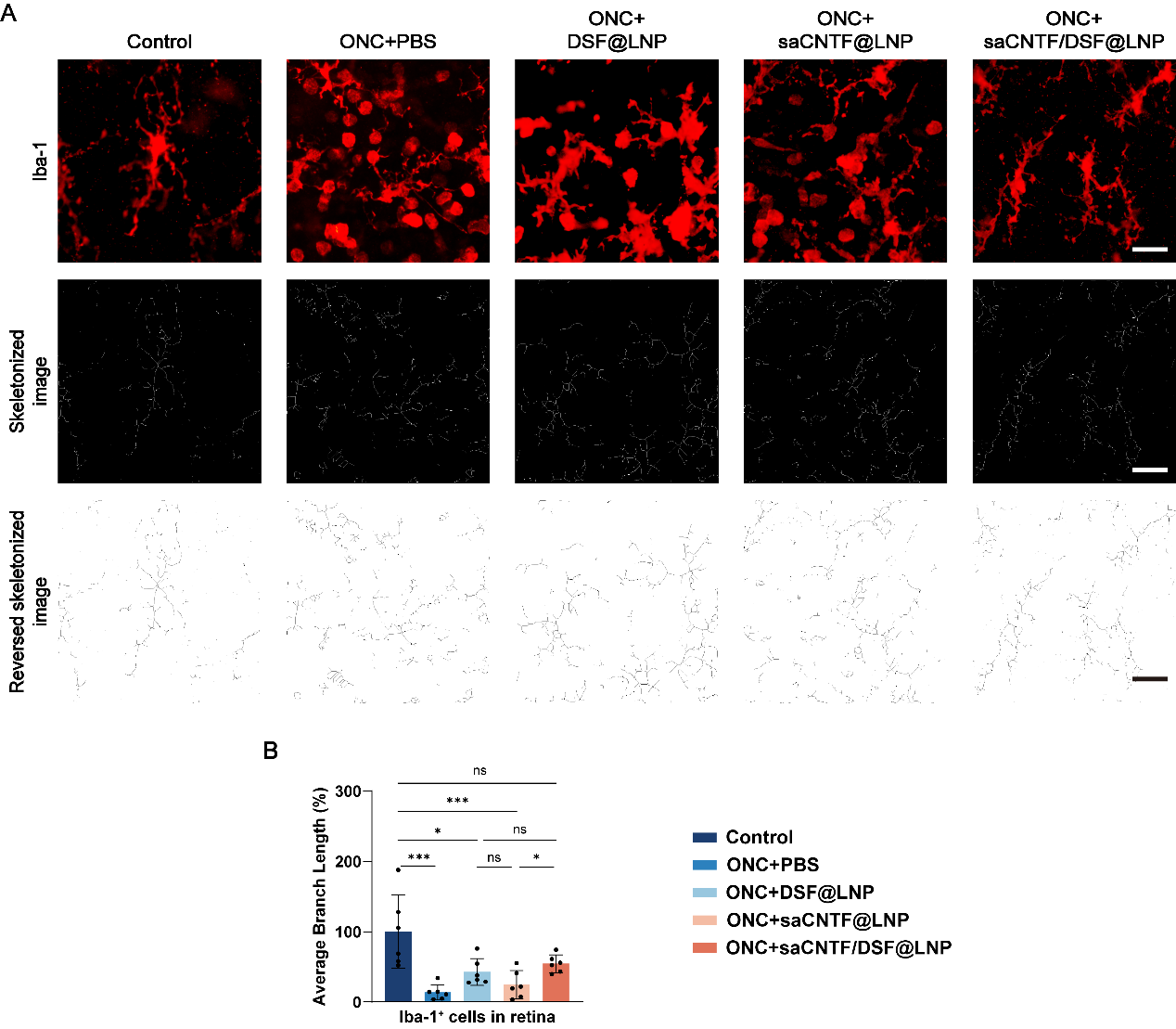
**

**Figure S21.** Analysis of microglial morphology in mouse retina via Iba-1 immunofluorescence staining. (A) Representative immunofluorescence images of Iba-1⁺ microglia across experimental groups, along with their corresponding skeletonized images processed using ImageJ, highlighting morphological details of microglial processes. Scale bar = 10 μm. (B) Average branch length of microglial cells across groups (*n*=6). Data are presented as mean ± SD; one-way ANOVA with Tukey’s multiple comparisons test, *P < 0.05, ns: the difference was not statistically significant.


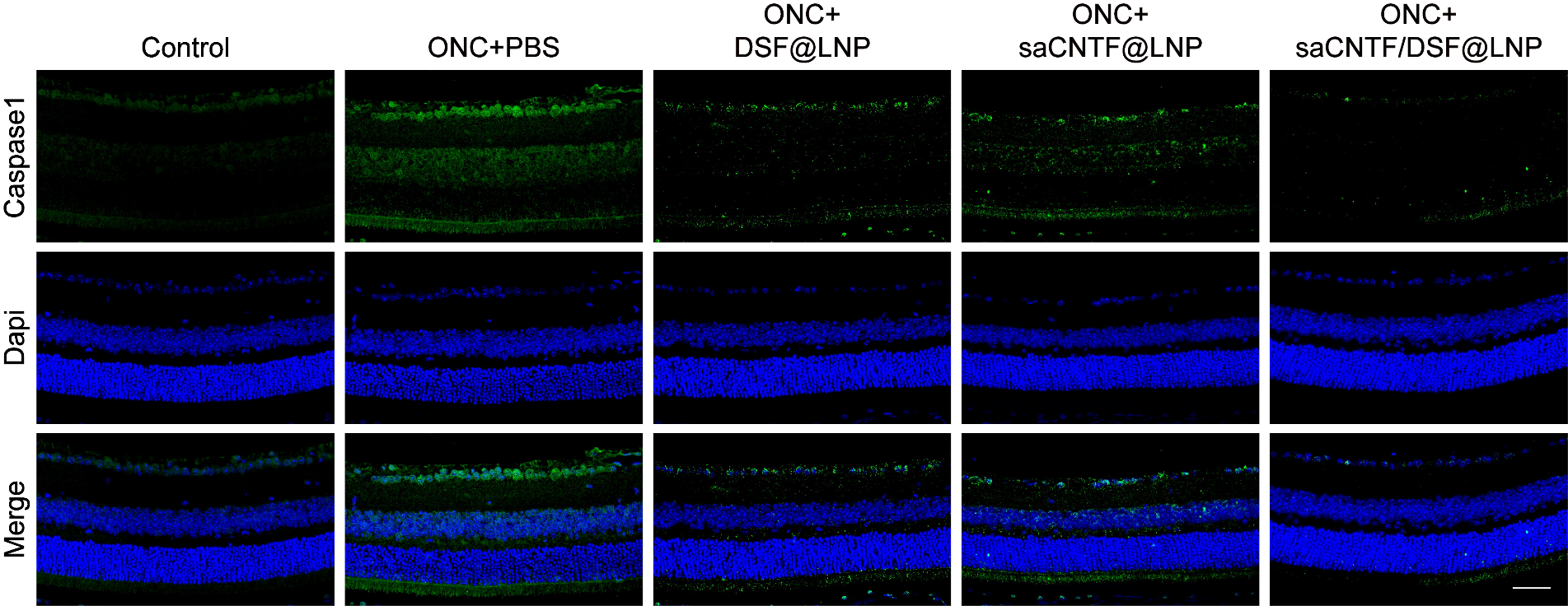


**Figure S22.** Immunofluorescence staining was used to observe the expression of caspase-1 in the retina tissue. Scale bars = 100 μm.


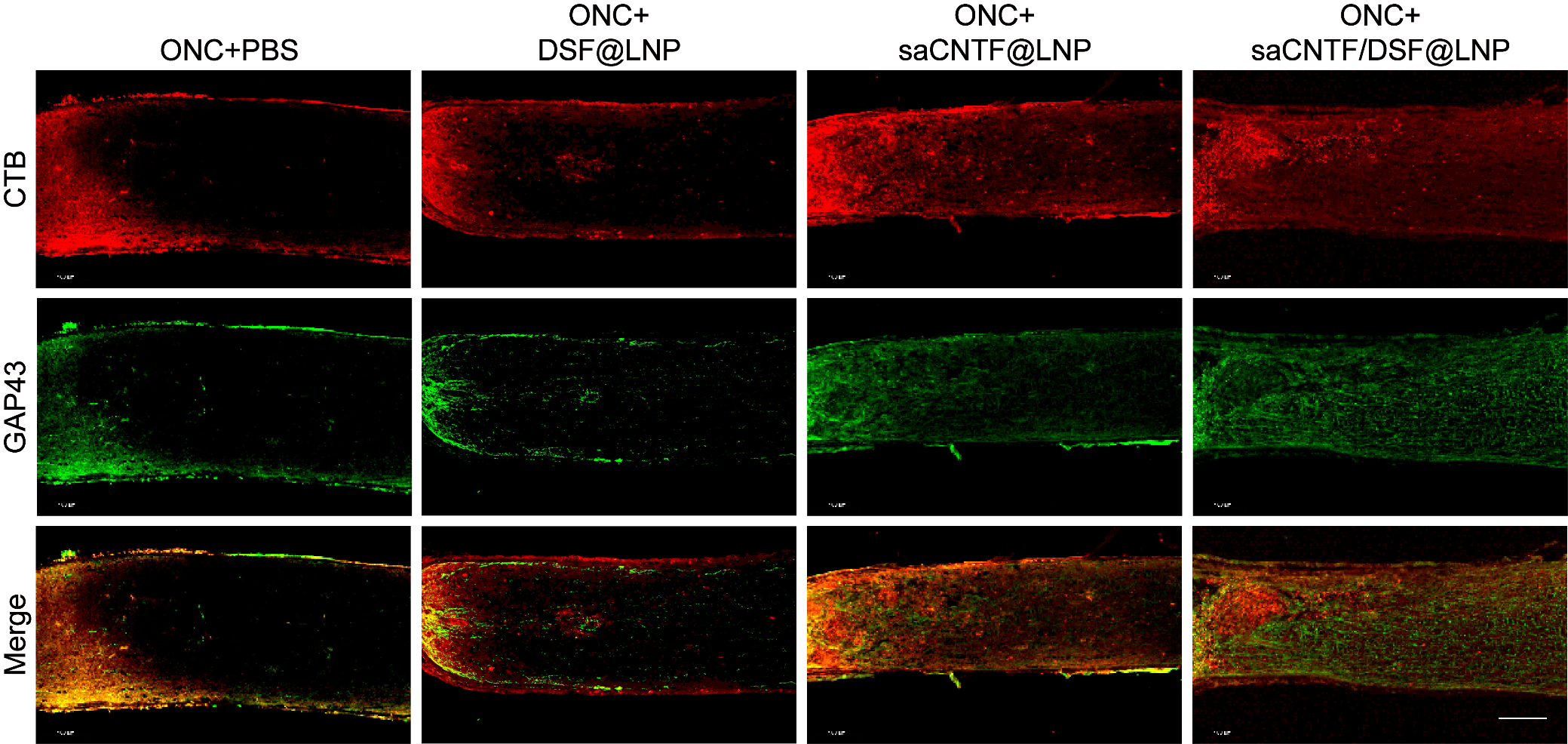


**Figure S23.** CTB fluorescence images and GAP43 immunofluorescence staining images of axons distal to the injury site. In the saCNTF/DSF@LNP group, there were axons labeled with both CTB (red) and GAP43 (green) distal to the injury site on days 28. The number of regenerated axons beyond the injury site in the saCNTF/DSF@LNP group was higher than that in the ONC, DSF@LNP or saCNTF@LNP group. Scale bar = 100 μm.


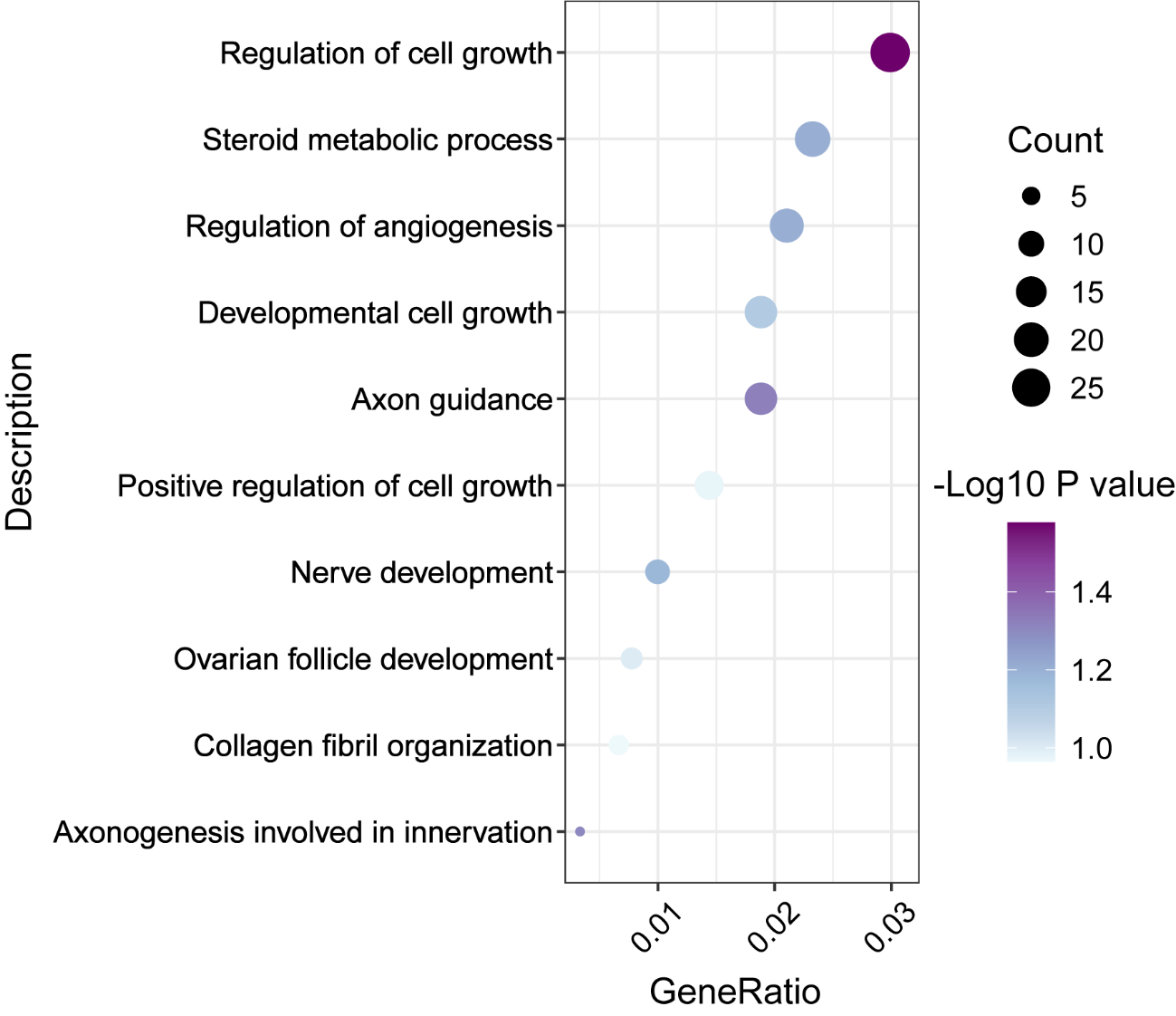


**Figure S24.** Gene Ontology enrichment analysis of differentially expressed genes between the saCNTF/DSF@LNP and Nigericin groups.

**
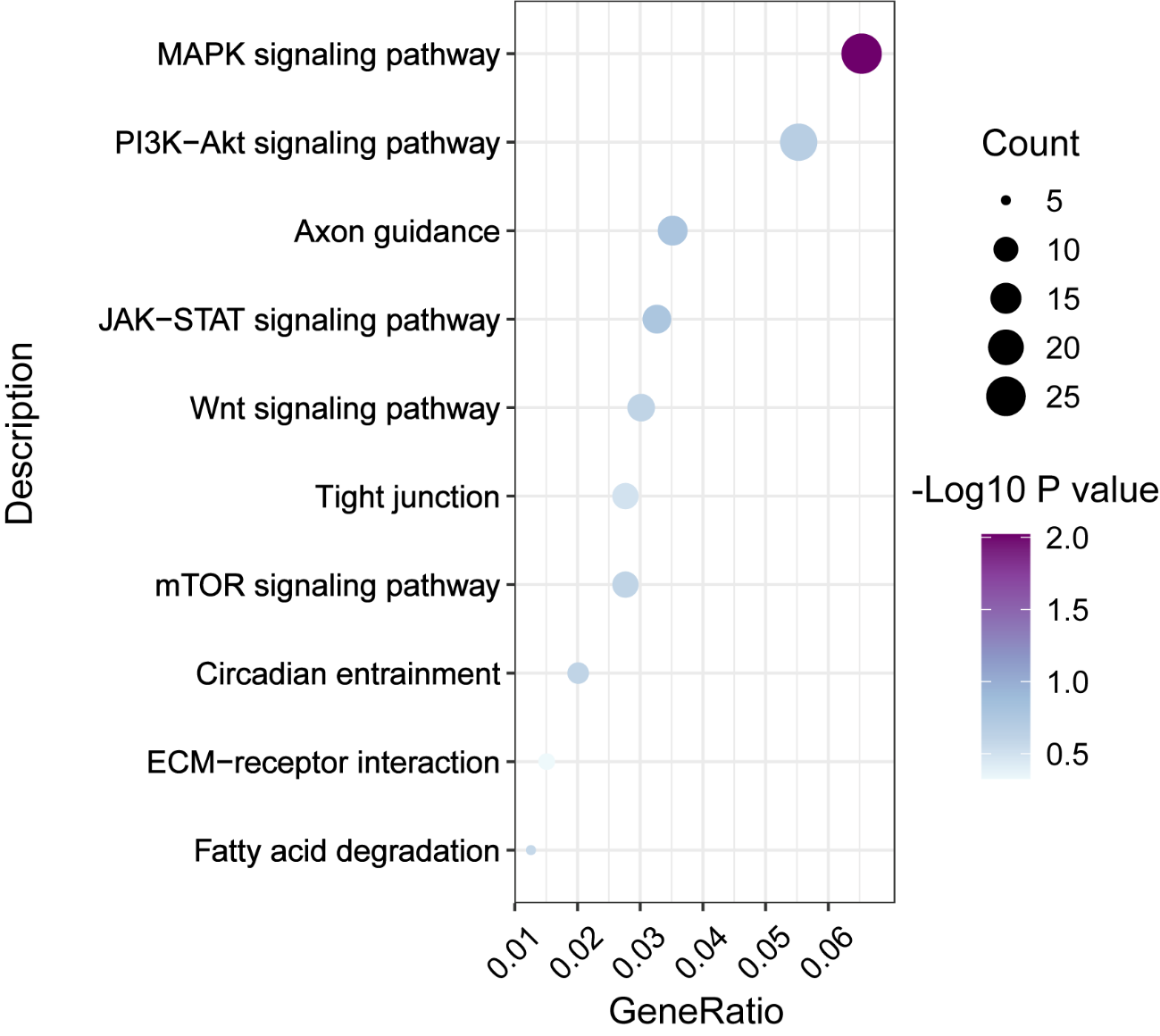
**

**Figure S25.** KEGG enrichment analysis of differentially expressed genes between the saCNTF/DSF@LNP and Nigericin groups.


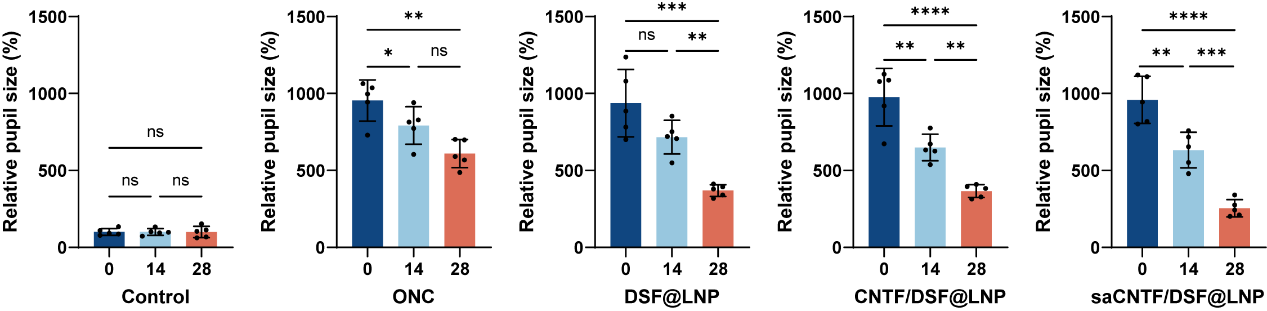


**Figure S26.** Quantification of pupil constriction (%) relative to pre-injury (Day 0) baseline at Days 14 and 28 post-ONC for the indicated groups (*n*=6).


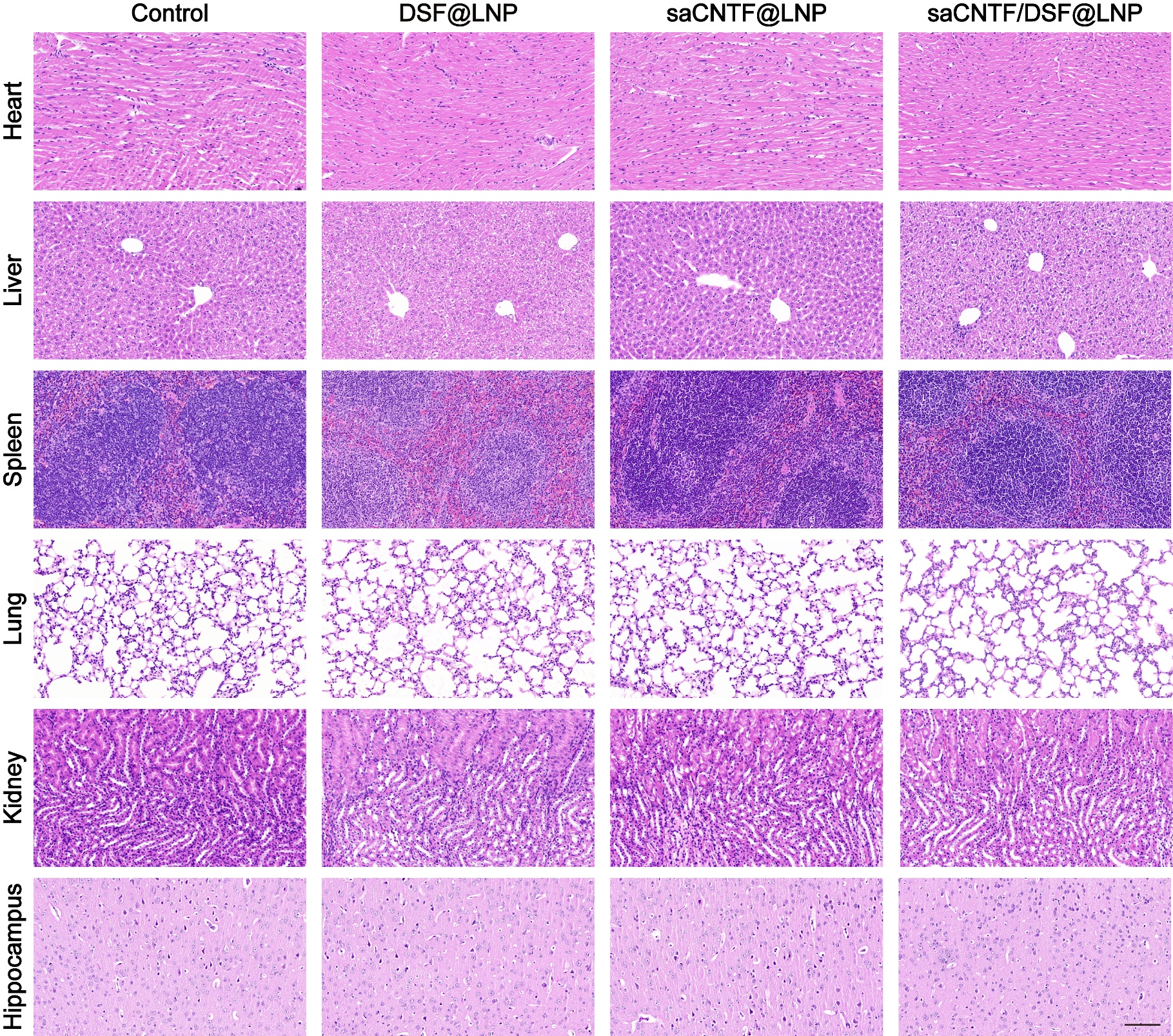


**Figure S27.**H&E-stained histological sections of the heart, liver, spleen, lungs, kidneys, and hippocampus. Scale bars = 100 μm.


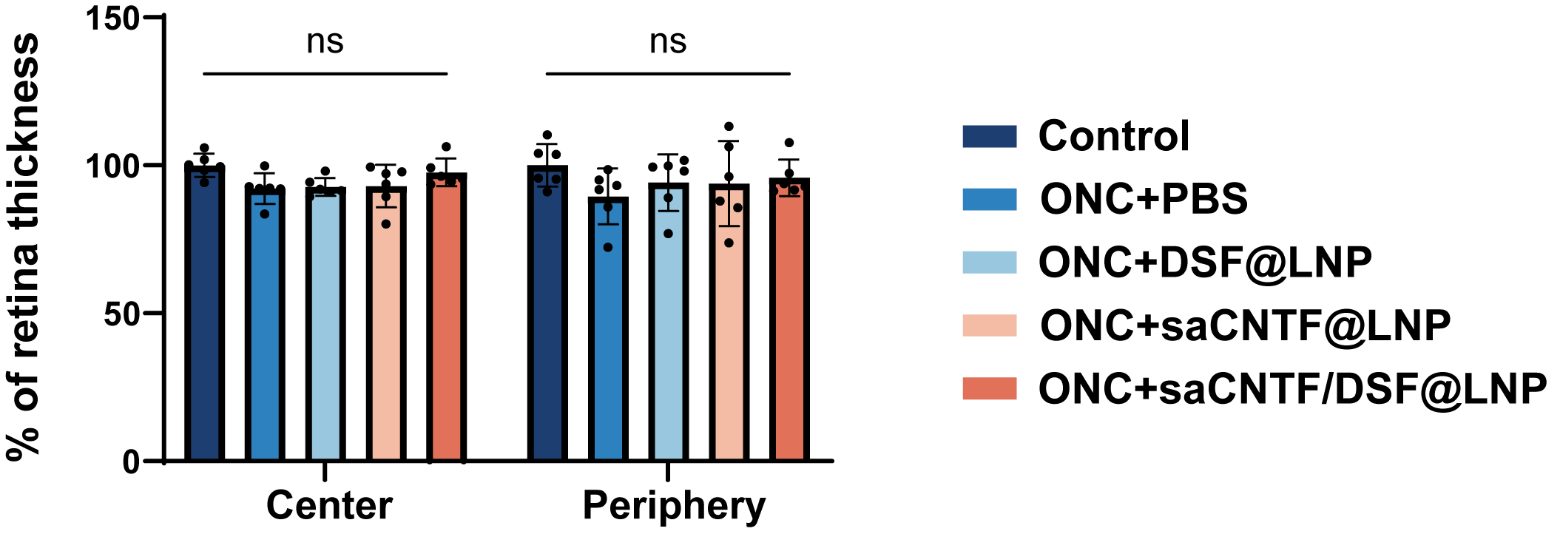


**Figure S28.** Statistical analysis of retinal thickness (*n*=6). Data are presented as mean ± SD; one-way ANOVA with Tukey’s multiple comparisons test, ns: the difference was not statistically significant.

**Figure S29.** Analysis of mouse blood routine data. The relative values of WBC, RBC, HGB, and PLT count in the saCNTF/DSF@LNP-treated group (*n*=4). Data are presented as mean ± SD; one-way ANOVA with Tukey’s multiple comparisons test, ns: the difference was not statistically significant.

**Table S1.** PCR primers used for qRT-PCR with SYBR-Green protocal

| Total mRNA expression | |
| --- | --- |
| Nlrp3-Mouse-F | TATCCACTGCCGAGAGGTGA |
| Nlrp3-Mouse-R | TCTTGCACACTGGTGGGTTT |
| Caspase1-Mouse-F | CCTGTCAGGGGCTCACTTTT |
| Caspase1-Mouse-R | TCCAAGTCACAAGACCAGGC |
| Gsdmd-Mouse-F | GCGTGTGACTCAGAAGACCT |
| Gsdmd-Mouse-R | AAACAGGTCATCCCCACGA |
| IL-1β-Mouse-F | TCGCAGCAGCACATCAACAAGAG |
| IL-1β-Mouse-R | AGGTCCACGGGAAAGACACAGG |
| Housekeeping gene |  |
| β-actin-Mouse-F | GTGACGTTGACATCCGTAAAGA |
| β-actin-Mouse-R | GCCGGACTCATCGTACTCC |

**Table S2.** Mouse CNTF Sequence

| Name | Sequence |
| --- | --- |
| Mouse CNTF | ATGGCCTTTGCTGAGCAGTCTCCATTGACTCTGCACCGGAGGGACCTGTGCTCCAGAAGCATCTGGCTGGCTCGAAAGATCCGGAGCGACTTGACCGCTCTCATGGAGTCTTATGTGAAACACCAGGGACTGAACAAGAACATCTCTCTCGACAGTGTTGACGGAGTTCCAGTGGCCTCCACTGACAGATGGAGCGAGATGACCGAGGCCGAAAGGCTGCAAGAGAACCTGCAGGCATACCGGACTTTCCAGGGTATGCTGACTAAGCTGCTGGAGGACCAGAGAGTGCACTTCACTCCAACAGAAGGTGATTTCCACCAGGCTATCCATACACTGACACTGCAGGTGAGTGCTTTCGCATACCAGTTGGAGGAGCTGATGGCACTGCTGGAGCAGAAAGTTCCAGAGAAAGAGGCTGACGGAATGCCAGTGACAATCGGCGATGGCGGCTTGTTCGAGAAGAAGCTGTGGGGACTCAAGGTACTGCAAGAGCTGAGCCAGTGGACAGTGCGAAGCATCCACGATCTGAGAGTGATCAGCAGTCACCACATGGGCATCAGCGCACACGAGTCTCACTATGGCGCCAAGCAGATGGATTATAAAGACCACGATGGTGACTATAAAGATCACGACATCGACTACAAAGACGATGACGATAAGTGA |
